# Novel markers for differentiation of lobular and ductal invasive breast carcinomas by laser microdissection and microarray analysis

**DOI:** 10.1186/1471-2407-7-55

**Published:** 2007-03-27

**Authors:** Gulisa Turashvili, Jan Bouchal, Karl Baumforth, Wenbin Wei, Marta Dziechciarkova, Jiri Ehrmann, Jiri Klein, Eduard Fridman, Jozef Skarda, Josef Srovnal, Marian Hajduch, Paul Murray, Zdenek Kolar

**Affiliations:** 1 Laboratory of Molecular Pathology, Institute of Pathology, Palacky University, Olomouc, Czech Republic; 2 Cancer Research U.K. Institute of Cancer Studies, University of Birmingham, UK; 3 Laboratory of Experimental Medicine, Department of Pediatrics, Palacky University, Olomouc, Czech Republic; 4 Department of Surgery, Palacky University, Olomouc, Czech Republic; 5 Department of Pathology, Tel Aviv University, Chaim Sheba Medical Center and Sackler School of Medicine, Tel Aviv, Israel

## Abstract

**Background:**

Invasive ductal and lobular carcinomas (IDC and ILC) are the most common histological types of breast cancer. Clinical follow-up data and metastatic patterns suggest that the development and progression of these tumors are different. The aim of our study was to identify gene expression profiles of IDC and ILC in relation to normal breast epithelial cells.

**Methods:**

We examined 30 samples (normal ductal and lobular cells from 10 patients, IDC cells from 5 patients, ILC cells from 5 patients) microdissected from cryosections of ten mastectomy specimens from postmenopausal patients. Fifty nanograms of total RNA were amplified and labeled by PCR and *in vitro *transcription. Samples were analysed upon Affymetrix U133 Plus 2.0 Arrays. The expression of seven differentially expressed genes (*CDH1, EMP1, DDR1, DVL1, KRT5, KRT6, KRT17*) was verified by immunohistochemistry on tissue microarrays. Expression of *ASPN *mRNA was validated by *in situ *hybridization on frozen sections, and *CTHRC1, ASPN *and *COL3A1 *were tested by PCR.

**Results:**

Using GCOS pairwise comparison algorithm and rank products we have identified 84 named genes common to ILC versus normal cell types, 74 named genes common to IDC versus normal cell types, 78 named genes differentially expressed between normal ductal and lobular cells, and 28 named genes between IDC and ILC. Genes distinguishing between IDC and ILC are involved in epithelial-mesenchymal transition, TGF-beta and Wnt signaling. These changes were present in both tumor types but appeared to be more prominent in ILC. Immunohistochemistry for several novel markers (EMP1, DVL1, DDR1) distinguished large sets of IDC from ILC.

**Conclusion:**

IDC and ILC can be differentiated both at the gene and protein levels. In this study we report two candidate genes, asporin (*ASPN*) and collagen triple helix repeat containing 1 (*CTHRC1*) which might be significant in breast carcinogenesis. Besides E-cadherin, the proteins validated on tissue microarrays (EMP1, DVL1, DDR1) may represent novel immunohistochemical markers helpful in distinguishing between IDC and ILC. Further studies with larger sets of patients are needed to verify the gene expression profiles of various histological types of breast cancer in order to determine molecular subclassifications, prognosis and the optimum treatment strategies.

## Background

Invasive ductal (IDC) and lobular carcinomas (ILC) are the most common histological types of breast cancer [1,2]. The terminology of ductal and lobular carcinomas is considered to be controversial as on purely anatomical grounds there is no justification for this nomenclature. Both carcinomas are derived from the terminal duct lobular unit (TDLU), and the differences in their morphology are likely to reflect the differences in mechanisms of carcinogenesis rather than the anatomical origin of the lesions. At presentation the clinical pathological parameters such as tumor site, size, grade and stage are similar for both tumor types [3], however, clinical follow-up data and the patterns of metastasis suggest that their development and progression are different [4,5]. Treatment for stage-matched tumors is similar [6], but ILCs are often resistant to neoadjuvant therapy [7]. Although patients with ILCs are older, have low grade tumor and less lymphatic invasion, they have no survival advantage compared with IDCs [8,9].

Expression profiling using microarrays is a powerful technology which enables the simultaneous study of the expression of thousands of genes and, in conjunction with laser capture microdissection, the high-throughput genetic analysis of morphologically distinct cell subpopulations within tumor tissue [10,11]. Microarray analysis has a number of applications, including tumor classification, molecular pathway modeling, functional genomics, and comparison of gene expression profiles between groups [12]. The study of gene expression in primary breast tumor tissues is complicated for two major reasons. First, breast cancer consists of many different cell types, including normal epithelial, stromal, adipose and endothelial cells. Second, tumor cells are morphologically and genetically diverse [13]. The recent development of laser capture microdissection has provided an opportunity to generate gene expression signatures from individual cell types [14-20].

Microarrays were used for analysis of breast tumor subclasses with clinical implication [21,22], for analysis of gene expression changes in single breast cancer cells from within the same tumor [14], for expression analysis of different gene families in breast cancer [23,24], and for analysis of gene expression in different cellular and tumor types [25-28]. ER status of the tumor was the most important discriminator of expression subtypes. Unsupervised hierarchical clustering segregated these tumors into two main clusters based on their basal (predominantly ER negative) and luminal (predominantly ER positive) characteristics [29]. Ductal breast cancer classes have been identified with aggressive phenotype and poor prognosis versus those with good prognosis [30-32]. Another study reported distinct expression patterns based on BRCA1 and BRCA2 status [33].

To date, few papers have been published on gene expression profiles of normal cell populations in the mammary gland [34]. Several studies suggest differences in expression profiles of IDC and ILC. Inactivating mutations of E-cadherin gene are very frequent in ILC [35]. However, the loss of E-cadherin expression was shown to be an independent prognostic marker for recurrence, especially in node-negative breast cancer patients, irrespective of the histological type [36]. Abnormal cytoplasmic and nuclear localization of p120, a member of the E-cadherin/catenin adhesion complex, is mediated by E-cadherin loss and occurs in the early stages of ILC [37]. Non-microdissected IDC and ILC tissues have also been used to study specific gene expression profiles of lobular and ductal tumors [38,39]. We aimed to identify genes differentially expressed between normal cell types (ductal and lobular), between tumor cells and normal cells as well as between tumor types (ductal no special type and lobular). Despite examining limited number of patients, our study is the first full genome analysis of microdissected ductal and lobular tumor and normal cells which allowed us to detect normal mammary epithelium- and cancer-specific genes.

## Methods

### Laser capture microdissection and RNA isolation

Altogether ten surgical specimens with either invasive ductal carcinoma NST (no special type) (n = 5) or invasive lobular carcinoma (n = 5) were investigated. This research protocol was approved by the ethics committee at Palacky University. Tumor and normal tissues from the same mammary gland were identified by an experienced pathologist, snap-frozen in liquid nitrogen and stored at -80°C for further analysis. For microdissection, frozen tissues were embedded in TissueTek medium and cut on standard cryostat (Leica CM1850, Leica Microsystems GmbH, Wetzlar, Germany). Frozen sections (7 μm) were immediately fixed in acetone, stained by hematoxylin and dehydrated in alcohol and xylene. All solutions were prepared using diethyl pyrocarbonate-treated water. RNase free instruments and RnaseZap (Sigma, St Louis, MO, USA) were used throughout.

At least 1000 infiltrating ductal or lobular tumor cells together with normal lobular and ductal cells were microdissected from cryosections using the Veritas™ Laser Capture Microdissection System (Arcturus Bioscience, Inc., Mountain View, CA, USA) according to standard protocols. When microdissecting normal cells, no attempt has been made to exclude basal or myoepithelial cells for two reasons. First, both ducts and lobules are atrophic in menopause, and no clearly defined layers of luminal and myoepithelial cells are preserved. Second, it is impossible to exclude contamination with or entrapment of myoepithelial cells during the microdissection procedure. Caps with captured cells were directly placed in 100 μl lysis buffer (Qiagen, Hilden, Germany). Total cellular RNA was isolated (RNeasy^® ^Micro Kit, Qiagen) according to manufacturer's recommendations and subsequently quantified on a Nanodrop spectrophotometer (Nanodrop Technologies, Wilmington, DE, USA).

### RNA amplification, microarray target synthesis and hybridization

Fifty nanograms of total RNA were reverse transcribed and amplified by Microarray Target Amplification Kit (Roche diagnostics, Basel, Switzerland). In brief, total RNA (50 ng) was converted into cDNA using a modified oligo (dT) primer (TAS-T7 Oligo (dT)_24_). A unique Target Amplification Sequence (TAS) with no homology to any known sequences in public databases generated the 3' anchor on the cDNA for subsequent PCR amplification. In order to include a 5' anchor sequence on the cDNA, the TAS-(dN)_10 _primer was used for the initiation of the second strand cDNA synthesis. After purification using the Microarray Target Purification Kit (Roche), PCR was performed using the TAS primer and Expand PCR Enzyme Mix which is optimized for long (>1 kb) and unbiased PCR products. In order to ensure that messages were not amplified to saturation, the optimal number of PCR cycles was estimated by preliminary PCR and agarose electrophoresis of PCR products from cycles 21, 24, 27, 30 and 33. The optimal number of PCR cycles was set either to 27 cycles for patients 3, 4, 8, 9, and 10 or 29 cycles for patients 1, 2, 5, 6, and 7. All three populations from each patient were amplified by the same number of PCR cycles. If higher PCR cycling was needed for any cell population, new microdissection, RNA isolation, cDNA synthesis and PCR amplification were performed until the same PCR cycling was possible. After purification with the Microarray Target Purification Kit (Roche), the PCR products were labeled with biotin-14-CTP (Invitrogen, CarlsBad, CA, USA) and biotin-16-UTP (Roche) by *in vitro *transcription using Microarray RNA Target Synthesis Kit T7 (Roche). The labeled cRNA was purified using the Microarray Target Purification Kit (Roche), quantified by spectrophotometer and checked by agarose electrophoresis. The entire amplification and labeling process was monitored by GeneChip^® ^Eukaryotic Poly-A RNA Control Kit (Affymetrix, Santa Clara, CA, USA) with exogenous positive controls which were spiked into the total RNA before cDNA synthesis. In all cases, 25 μg of each biotinylated cRNA preparation was fragmented, assessed by gel electrophoresis, and placed in hybridization cocktail containing biotinylated hybridization controls (GeneChip™ Eukaryotic Hybridization Control Kit, Affymetrix). Samples were first hybridized to Test3 Arrays for 16 hours, washed, stained using antibody-mediated signal amplification and scanned. After passing this quality control stage, the samples were hybridized onto the large Human Genome U133 Plus 2.0 Arrays (Affymetrix).

### Microarray data analysis

Scanned images of microarray chips were analysed by the GCOS (GeneChip Operating Software) from Affymetrix with the default settings except that the target signal was set to 100. Differentially expressed genes between cell types were identified using the GCOS change algorithm and Rank Products (RP) [40] following RMA (Robust Multiarray Analysis) [41]. GCOS pairwise analysis was performed to compare gene expression levels among the normal lobular, normal ductal and tumor cells within individual patients and between lobular and ductal carcinoma cells of different patients. For each comparison between two cell types, the number of increase and decrease calls of each probe set was calculated using MS Excel and probe sets with the highest number of consistent changes among all patients were identified. Probe level quantile normalization [40] and robust multiarray analysis [41] on the raw. CEL files were performed using the Affymetrix package of the Bioconductor [42]. The cutoff value of percentage of false-positives for RP analysis was set to 10%. Gene lists were uploaded to DAVID (Database for Annotation, Visualization and Integrated Discovery) [43], and functional annotation was performed. Further informations on genes were obtained from public databases, such as Gene Cards [44] and NETAFFX Analysis Center [45]. Hierarchical clustering was performed using dChip software [46]. The data discussed in this publication have been deposited in NCBI Gene Expression Omnibus (GEO) [47], and are accessible through GEO Series accession number GSE5764.

### Validation by immunohistochemistry

Seven differentially expressed genes were verified by immunohistochemical staining on tissue microarrays (TMA), which were constructed from 119 breast cancer cases and contained 278 cores of 2.0 mm diameter. They consisted of 80 ductal carcinomas, 29 lobular carcinomas, one tubular carcinoma, 3 medullary carcinomas, 2 tubular-lobular carcinomas, 2 mixed ductal-lobular carcinomas, one mucinous and one papillary carcinoma. The construction of the tissue microarrays was done using a tissue arrayer (Beecher Instruments, Inc., Sun Prairie, WI, USA) [48]. The paraffin sections from 22 additional breast cancer samples containing normal mammary gland structures were used for comparison.

TMA sections were stained using standard immunohistochemical methods. Monoclonal mouse antibodies against cytokeratin 17 (dilution 1:100, clone 2D4-1G9, Abnova, Taipei, Taiwan), cytokeratin 5/6 (dilution 1:100, clone D5/16 B4, Dako, Denmark) and E-cadherin (dilution 1:50, clone NCH-38, Dako), polyclonal mouse antibodies against EMP1 (epithelial membrane antigen 1; dilution 1:100, clone 2D4-1G9, Abnova), DDR1 (discoidin domain receptor 1; dilution 1:100, Abnova) and DVL1 (human homolog of the Drosophila dishevelled gene; dilution 1:100, Abnova) were used. Microwave antigen retrieval was performed in citrate buffer (pH 6.0). For antigen visualization, the EnVision/HRP system and DAB+ (Dako) were used, slides were subsequently counterstained with hematoxylin, dehydrated and mounted in Canadian balsam. Immunohistochemical procedure was optimized by testing different antigen retrieval methods and negative controls. We regarded cells as immunoreactive when an obvious membranous or submembranous (E-cadherin, DDR1, EMP1) and cytoplasmic (cytokeratins, DVL1) staining was seen. Immunoreactivity was scored as follows: retained (++) when more than 50% of membrane/cytoplasm were strongly positive, reduced (+) when 10–50% of the membrane/cytoplasm were positive, and absent (-) when 0–10% of the membrane/cytoplasm were positive. Frequencies of positive and negative staining in tumor and normal tissues were compared by Fisher's exact test (Statistica 6.0, StatSoft, Prague, Czech Republic).

### Validation by *in situ *hybridization

Asporin mRNA expression was verified by chromogenic *in situ *hybridization (CISH) on frozen sections. Fluorescein labeled oligonucleotide (5'AAG TTG GTG GTA AGC CTT TAG GAA CTG AGG-FAM) was designed to the junction of exons five and six of the asporin mRNA (Generi Biotech, Hradec Kralove, Czech Republic). Sections were mounted on Superfrost Plus slides and stored at -80°C until further staining. Before CISH, the sections were heated at 50°C for 2 min, and then dried at room temperature for 30 min. Tissues were delipidized in chloroform at room temperature for 5 min and dried to evaporate the chloroform. Block of endogenous peroxidase activity was followed by incubation of tissue sections in 10% paraformaldehyde for 7 min, then washed in PBS for 3 min and in 2× SSC (1× SSC contains 150 mM NaCl, 15 mM sodium citrate; pH 7.2) twice for 5 min. The sections were covered with 100 μl hybridization buffer (60% formamide, 5× SSC) containing 150 ng/ml of probe and incubated in moist chamber at 37°C for 22 hours. After the hybridization, unbound probe was washed from the sections at 37°C by 2× SSC for 5 min, then three times with 60% formamide in 0.2× SSC at 37°C for 5 min, and twice with 2× SSC at room temperature for 5 min. Then polyclonal rabbit anti-FITC/HRP (dilution 1:40, Dako) was applied at room temperature for 45 min, followed by washing in 2× SSC twice for 10 min. For visualization, DAB+ (Dako) was used, slides were subsequently counterstained with hematoxylin, dehydrated and mounted in Canadian balsam.

### Validation by PCR

Products after PCR amplification (see RNA amplification, microarray target synthesis and hybridization) were used for verification of *CTHRC1, ASPN *and *COL3A1 *gene expression using gene specific PCR. The primers were as follows: *CTHRC1 *forward 5'ACA AGT GCC AAC CCA GAT AGC AAC, reverse 5'ATC GCA CTT CTT CTG TGG AAG GAC (20 cycles of 95°C, 59°C and 72°C 1 min each, product length 79 bp, kindly provided by Dr. Danuta Radzioch, McGill University, Montreal, Canada), *ASPN *forward 5'GTT CAG CTT GGG AAC TTT GGA ATG TAA, reverse 5'ACT GCA ATA GAT GCT TGT TTC TCT CAA CCC (20 cycles of 95°C, 58.5°C and 72°C 1 min each, product length 243 bp [modified from [49]], and *COL3A1 *forward 5'TTG TCA ACC AGT GCA AGT GAC CGA C, reverse 5'TGG TGA GCA CAG TCA TTG CTC TGC A (20 cycles of 95°C, 59°C and 72°C 1 min each, product length 276 bp) [50]. PCR products were separated on 10% polyacrylamide gels, stained with ethidium bromide and captured using a gel documentation system (DIANA II with cooled CCD camera, Raytest, Germany). Band intensities, expressed as "OD (optical density) × cm", were assessed by Multi-Analyst densitometric software (Bio-Rad, USA). The results were correlated with fluorescence signals from the relevant Affymetrix probe sets by Spearman coefficient using Statistica 6.0 software (StatSoft, Czech Republic).

## Results

### Unsupervised clustering

**Figure 1:**
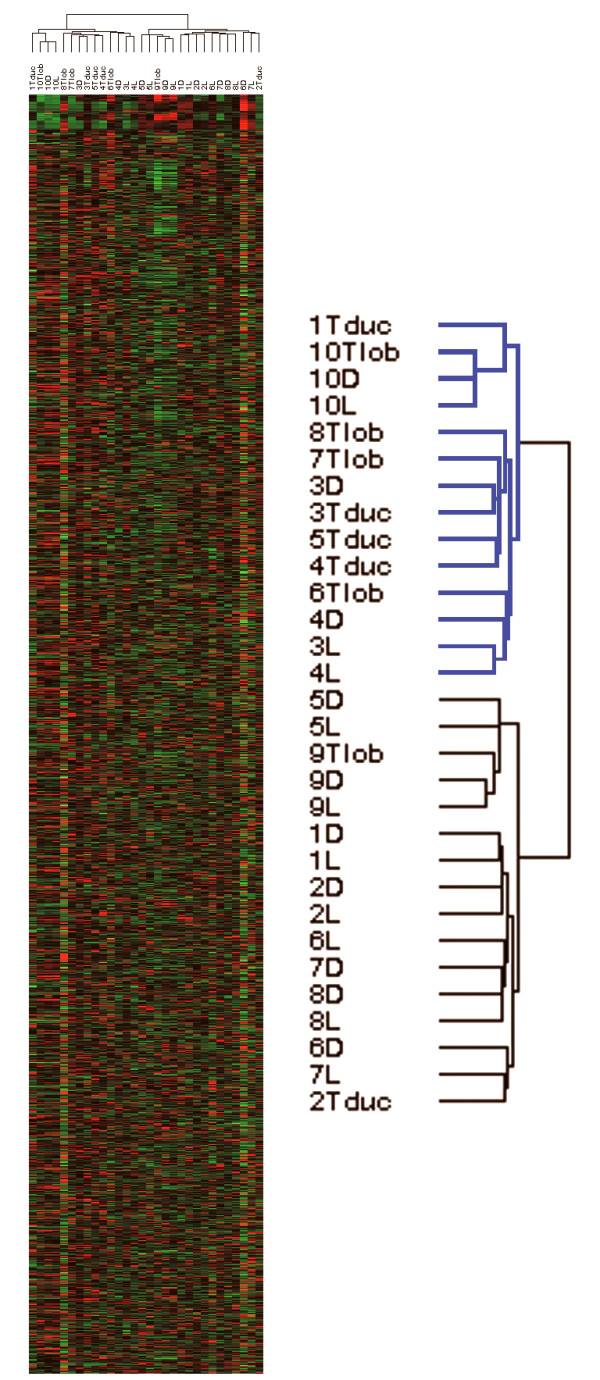
**Unsupervised hierarchical clustering of all samples using all probe sets**. No tumor types are grouped together, however, two large clusters are evident: one (blue) mainly consists of tumor cells and the other (black) mainly consists of normal cells. This suggests differences in global gene expression profiles of tumor and normal cells. 1–10, case numbers; D, ductal normal cells; L, lobular normal cells; Tduc, ductal tumor; Tlob, lobular tumor.

The clinical and histopathological characteristics including lymph node status, tumor grade, expression of estrogen/progesterone receptors (ER/PgR), c-erbB-B2/HER2/neu and accompanying lesions of ten breast cancer patients are shown in Table 1. We microdissected normal and tumor cells from these cases which resulted in a total of 30 samples for gene expression profiling (10 normal ductal, 10 normal lobular, 5 tumor ductal and 5 tumor lobular). Unsupervised clustering of all samples was performed using all probe sets without filtering. No tumor types were grouped together but two large clusters were evident: one mainly consisted of tumor cells and the other cluster mainly of normal cells. This clearly suggests differences in global gene expression profiles of tumor and normal cells (Figure 1).

### Genes differentially expressed between normal ductal and lobular cells

**Figure 2:**
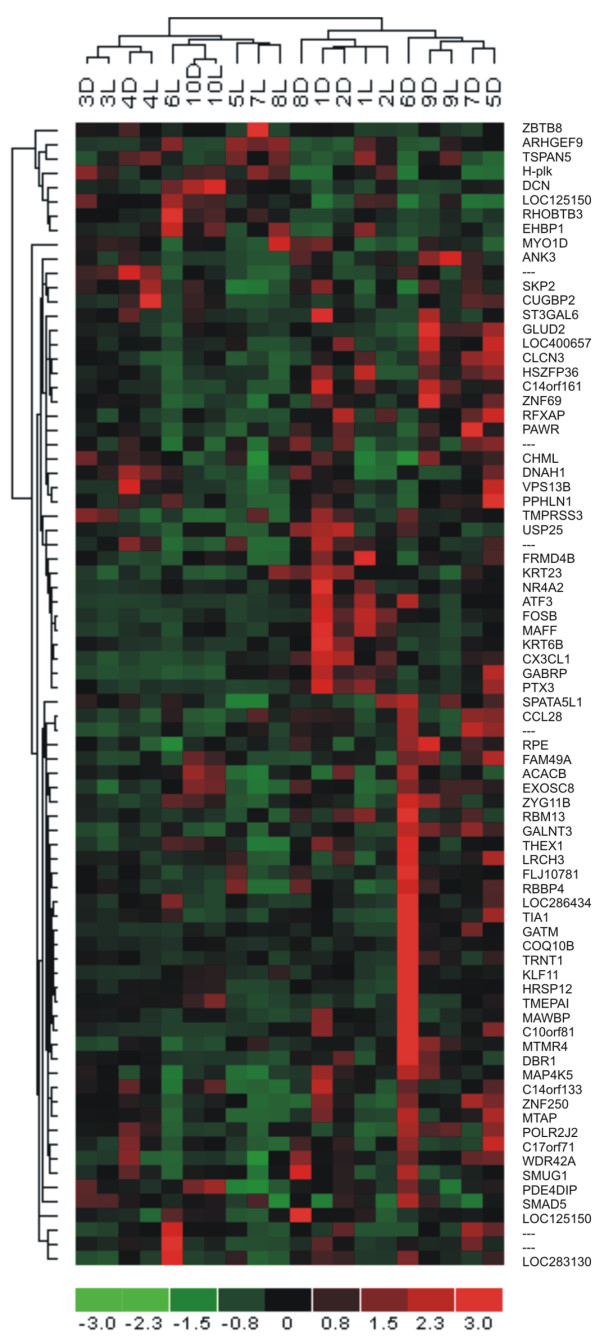
**Hierarchical clustering of normal ductal and lobular cells based on 82 probe sets found by both rank products and pairwise analysis**. Ductal and lobular cells from the same patients tend to cluster together, and they can not be well separated from each other. This suggests expected similarities of expression signatures in normal epithelial (ductal and lobular) cells of mammary gland tree. 1–10, case numbers; D, ductal normal cells; L, lobular normal cells.

In this comparison 367 probe sets were identified by pairwise comparison, and 501 probe sets by Rank Products (RP). There were 82 probe sets (78 named genes) identified by both methods. In pairwise comparisons, the differentially expressed genes were found by selecting those with count of changes more than 6 (out of 10, either increase or decrease). Multiple cytokeratins (5, 7, 14, 15, 17) were expressed in both cells, but none of them was useful to separate two cell types. Genes encoding proteins involved in proteolysis and metabolism (*USP25, TMPRSS3, ACACB*), protein biosynthesis and modification (*PDE4DIP, LOXL1*), ion transport (*CLCN3, GABRP*) and protein kinase cascade (*MAP4K5*) as well as genes regulating transcription (*RFXAP, HSZFP36*) were upregulated in ductal cells. However, genes regulating cell growth, activation and motility (*TSPAN5*), genes encoding actin-binding proteins (*EHBP1*), small leucine-rich proteoglycan (*DCN*), and proteins with GTPase activity (*RHOBTB3, ARHGEF9*) were overexpressed in lobular cells (Table 2). Hierarchical clustering of normal cell types based on the 82 probe sets found by both RP and pairwise comparison showed that gene expression profiles of normal ductal and lobular cells from the same patients were similar, and they could not be well separated from each other (Figure 2).

### Genes differentially expressed between tumor cell types and normal cells

A comparison of ductal carcinoma cells with normal ductal cells identified 1055 probe sets by pairwise analysis, 604 probe sets by RP and 326 probe sets by both methods. A comparison of ductal carcinoma cells with normal lobular cells identified 792 probe sets by pairwise analysis, 347 probe sets by RP and 171 probe sets by both methods. A comparison of lobular carcinoma cells with normal ductal cells identified 1022 probe sets by pairwise analysis, 350 probe sets by RP and 201 probe sets by both methods. A comparison of lobular carcinoma cells with normal lobular cells identified 983 probe sets by pairwise analysis, 344 probe sets by RP and 208 probe sets by both methods (see Additional file 1). In pairwise comparisons, the differentially expressed genes were identified by selecting those with count of changes more than 4 (out of 5, either increase or decrease).

#### Comparison between lobular carcinoma and normal cells

Combined pairwise analysis and RP have identified 106 differentially expressed probe sets (84 named genes) common to lobular carcinoma versus normal ductal and lobular cells. Of these probe sets, several genes encoding proteins involved in extracellular matrix (ECM)-tumor interaction and focal adhesion (*COL6A3, COL8A1, CTHRC1, THBS2, COMP*) were upregulated, whereas *ITGA2*gene with the same function was downregulated in tumor cells. *ASPN *(asporin) was one of the upregulated genes with the highest fold change. It encodes a protein with porin activity which belongs to a family of leucine-rich repeat (LRR) proteins associated with the cartilage matrix.

Of the differentially expressed genes involved in Wnt signaling, *SFRP2 *(secreted frizzled-related protein 2) and *WISP1 *(WNT1 inducible signaling pathway protein 1) were upregulated in lobular carcinomas. Several genes encoding proteins involved in actin, calcium and metal ion binding were downregulated (*MYBPC1, DST, PIP, CA2*), and some genes with the same function (*BGN, ADAM12*) were upregulated in lobular carcinomas. *CDH1 *(E-cadherin) was downregulated in lobular tumors accompanied by downregulation of *ITGA2 *(integrin, alpha 2) which it is a ligand of. Several other genes were differentially expressed between lobular carcinomas and normal cells. *LYZ/LILRB1 *was upregulated in lobular cancer cells. Other downregulated genes were as follows: *CGNL1 *is a cellular myosin that appears to play a role in cytokinesis, cell shape, and specialized functions such as secretion and capping; *EHF *encodes a protein that belongs to an ETS transcription factor subfamily characterized by epithelial-specific expression (ESEs). This protein may be involved in epithelial differentiation and carcinogenesis; *CLIC6 *encodes a member of the chloride intracellular channel family of proteins and is involved in ion transport (Table 3).

#### Comparison between ductal carcinoma and normal cells

Combined pairwise analysis and RP have identified 90 differentially expressed probe sets (74 named genes) common to ductal carcinoma versus normal ductal and lobular cells. In particular, transcription regulators (*ATF3*, *PIGR*), genes encoding proteins with cytokine and growth factor activity (*PTN, CX3CL1*) and genes encoding proteins involved in ion transport and metabolism (*ATP1A2*, *MMP7*) were downregulated in ductal carcinomas when compared with normal cells (Table 3).

#### Comparison between both types of tumor cells and normal cells

**Figure 3:**
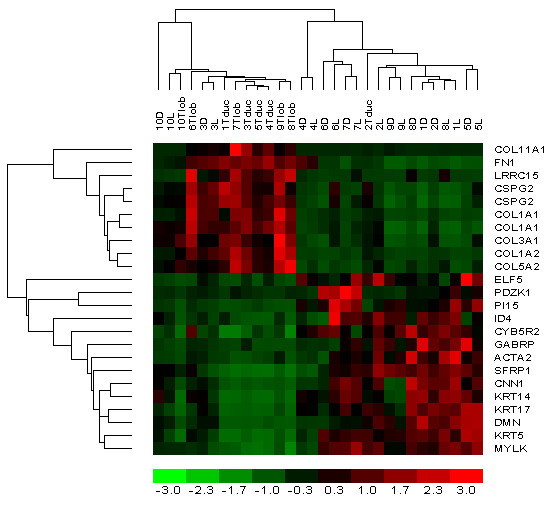
**Hierarchical clustering of tumor and normal samples based on 25 probe sets common to ductal and lobular carcinoma versus normal cells identified by both rank products and pairwise analysis**. Tumor samples are grouped together, however, gene expression profiles of normal cell types from cases 10 and 3 are different from those of other normal cells, and tumor cells from case 2 are different from those of other tumor cells. 1–10, case numbers; D, ductal normal cells; L, lobular normal cells; Tduc, ductal tumor; Tlob, lobular tumor.

Of 90 probe sets differentially expressed between ductal carcinoma and both normal cells, and 106 probe sets differentially expressed between lobular carcinoma and both normal cells, only 25 probe sets were common (Table 3). Hierarchical clustering based on those 25 probe sets showed that tumor samples were grouped together, however, gene expression profiles of normal cell types from cases 10 and 3 were different from other normal cells, and tumor cells from case 2 were different from other tumor cells (Figure 3).

Several upregulated genes (collagen type I, III, V, XI, fibronectin 1, versican) were related to tumor- ECM interactions and focal adhesion. However, another gene mediating focal adhesion, *MYLK*, was downregulated in tumor cells. This gene encodes a myosin light polypeptide kinase containing 1 fibronectin type-III domain. Genes encoding proteins involved in ion and electron transport (*CYB5R2*, *GABRP*), and genes encoding proteins with transcription factor and regulator activity (*ELF5, ID4*) were downregulated in both populations of tumor cells. They have also been implicated as regulators of cell proliferation, differentiation, and transformation. In addition, genes involved in cell differentiation and apoptosis (*PDZK1*) and genes encoding actin and actin-binding proteins (*CNN1*, *ACTA2*) were also downregulated in both types of tumor cells. Wnt signaling molecules were differentially expressed in our samples. Of these, *SFRP1 *(secreted frizzled-related protein 1) was downregulated in tumor cells, and genes involved in calcium regulation pathway were not significantly changed. Structural constituents of cytoskeleton such as type I (14, 15, 17, 23) and type II (5) keratins were downregulated in tumor cells. Several other genes were also differentially expressed between tumor and normal cells. *PI15 *(peptidase inhibitor 15) was downregulated, whereas *LRRC15 *(leucine rich repeat containing 15) was upregulated in both tumor cells.

### Genes differentially expressed between ductal and lobular carcinomas

**Figure 4:**
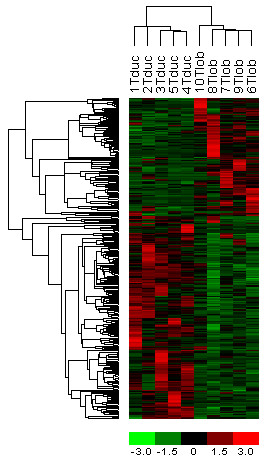
**Hierarchical clustering of invasive ductal and lobular breast carcinomas based on 325 probe sets identified by rank products and/or pairwise analysis**. Tumor types are well separated. 1–10, case numbers; Tduc, ductal tumor; Tlob, lobular tumor.

In this comparison 208 probe sets were identified by pairwise comparison, 122 probe sets by RP, and 32 probe sets (28 named genes) by both methods (Table 4). In pairwise comparisons, the differentially expressed genes were identified by selecting those with count of changes more than 19 (out of 25 inter-patient comparison, every ductal carcinoma against every lobular carcinoma, either increase or decrease). These tumors were well separated by hierarchical clustering based on all 325 probe sets identified by RP and/or pairwise analysis (Figure 4). The expression of genes encoding proteins involved in cell adhesion was changed. Although *CDH1 *(E-cadherin), a classical member of the cadherin superfamily, was downregulated, *THBS4 *encoding calcium-binding adhesive glycoprotein, thrombospondin-4, was upregulated in all lobular carcinomas. *DDR1 *encoding receptor tyrosine kinase was overexpressed in ductal carcinomas.

The *DVL1 *gene encoding protein involved in Wnt signaling and the leucine-rich repeat protein *ASPN *(asporin) were upregulated in lobular carcinomas. Ductal carcinomas showed upregulated genes which are involved in cell proliferation, signaling and cell cycle regulation, including *RHOU*, member of the Rho family of GTPases stimulating quiescent cells to reenter the cell cycle; *PCSK6 *encoding a calcium-dependent serine endoprotease; *PRKCI *encoding calcium-independent and phospholipid-dependent protein kinase C; *PPP3CB *encoding protein phosphatase 3; and *CKS2 *encoding a component of the CDC28 protein kinase. Epithelial membrane protein 1 (*EMP1*) was the only upregulated gene involved in cell growth and proliferation in lobular carcinomas. These changes were accompanied by the differential expression of transcription regulators. Majority of genes with this function were upregulated in ductal carcinomas such as *AHCTF1, IRAK1, NRIP1, ADNP*. Overexpressed genes in lobular carcinomas were the tumor suppressor *FOXP1 *and another transcription regulator *MID1*.

The genes encoding proteins involved in ubiquitin-mediated proteolysis, such as *USP3, RKHD2 *and *TTC3*, and nuclear components, such as *DTL, GLCC11, TTC14, FAM54A, HIST1H3B*, were upregulated in ductal carcinomas. Majority of genes encoding proteins with enzyme activity or implicated in metabolism were also upregulated in ductal carcinomas (*STK4, SLC1A2, B3GALT3, OSBPL10, CRBN, CHML, YWHAB*).

Two lobular and three ductal carcinomas were estrogen receptor-negative, whereas three lobular and two ductal carcinomas were estrogen receptor-positive. Hierarchical clustering using all probe sets was performed to determine whether receptor-positive and receptor-negative tumors could be separated. The tumors of the same histological type showed similar gene expression profiles without differences in relation to ER status as well as to other clinical parameters such as nodal status, stage and the expression of other immunohistochemical markers (data not shown).

### Validation by immunohistochemistry on tissue microarrays

**Figure 5:**
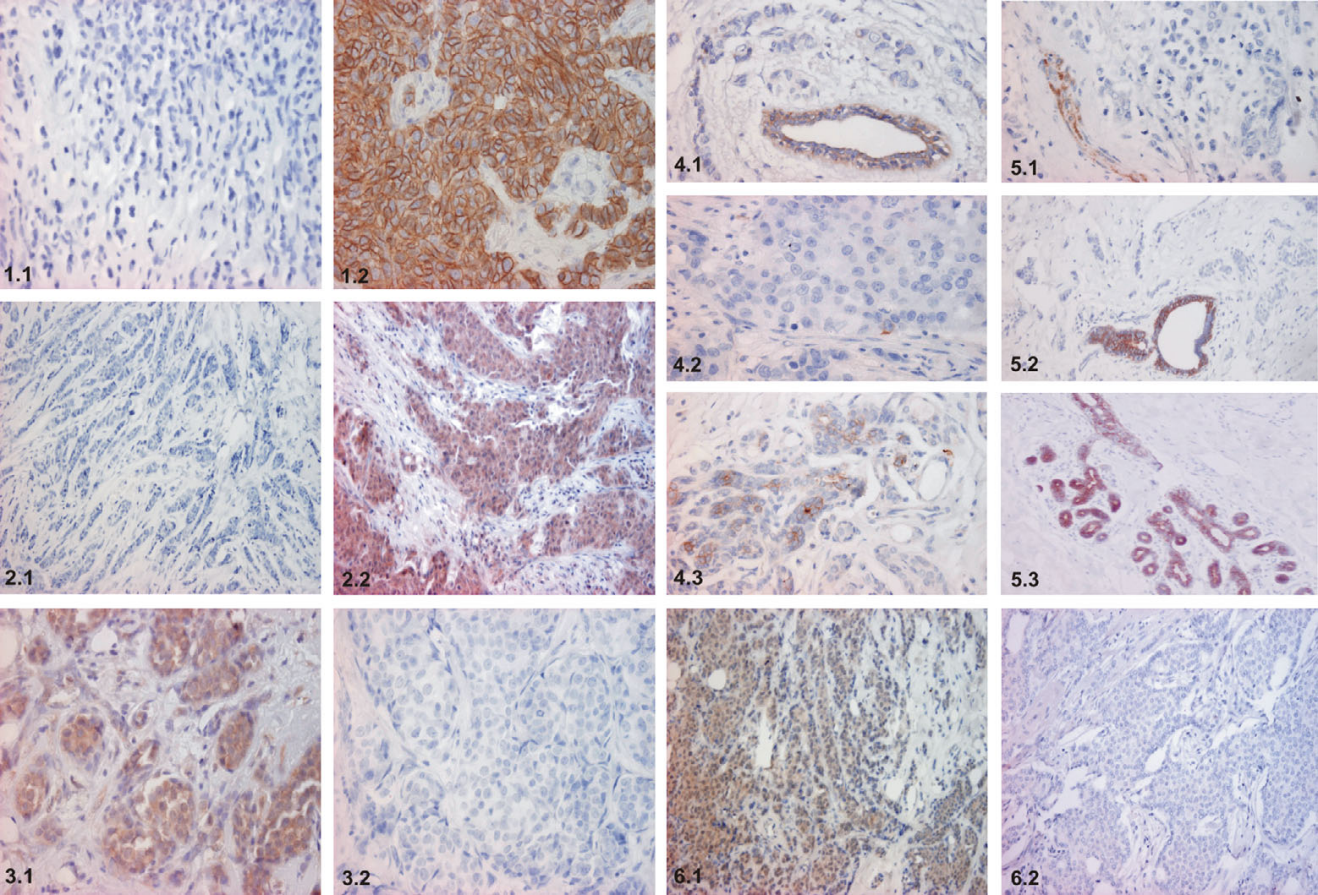
**Representative immunohistochemical staining for the selected proteins: E-cadherin, DDR1, DVL1, cytokeratin 5/6, cytokeratin 17 and EMP1**. 1.1. E-cadherin is negative in lobular carcinoma; 1.2. E-cadherin is positive in ductal carcinoma; 2.1. DDR1 is negative in lobular carcinoma; 2.2. DDR1 is positive in ductal carcinoma; 3.1. DVL1 is positive in lobular carcinoma; 3.2. DVL1 is negative in ductal carcinoma; 4.1. Cytokeratin 5/6 is negative in lobular carcinoma cells, but its expression is retained in normal ductal epithelial cells; 4.2. Cytokeratin 5/6 is negative in ductal carcinoma cells; 4.3. Duct lobular unit in normal mammary gland tissue is positive for cytokeratin 5/6; 5.1. Cytokeratin 17 is negative in lobular carcinoma cells, but its expression is retained in normal ductal epithelial cells; 5.2. Cytokeratin 17 is negative in ductal carcinoma cells, but its expression is retained in normal ductal epithelial cells; 5.3. Duct lobular unit in normal mammary gland tissue is positive for cytokeratin 17; 6.1. EMP1 is positive in lobular carcinoma; 6.2. EMP1 is negative in ductal carcinoma.

Seven differentially expressed genes (*KRT5, KRT6 *and *KRT17 *between tumor and normal cells, *CDH1, EMP1, DDR1 *and *DVL1 *between lobular and ductal carcinomas) were verified by immunohistochemical detection of proteins on TMA slides comprising of cores from 119 cases. The clinical and histopathological characteristics of these patients are shown in Table 5. The reduced expression or absence of cytokeratins 5/6 and 17 (*KRT5, KRT6, KRT17*) was found in both tumor tissues in comparison to terminal duct lobular units in 22 normal mammary tissues (*p *< 0.0001) (Table 6 and Figure 5). In a majority of ducts and lobules including TDLU, these cytokeratins were expressed in both basal and luminal cells, verifying the previously described variability of the expression of basal cytokeratins and their relationship to the cellular origin [51]. Cytokeratin 5 and 17 have also been found in a subset of breast cancer and identified patients with poor clinical outcome [31].

E-cadherin (*CDH1*) has successfully separated ductal and lobular invasive carcinomas. It was absent in 93.3% of lobular tumors compared with only 15% of ductal tumors (*p *< 0.0001). Epithelial membrane protein 1 (*EMP1*), discoidin domain receptor 1 (*DDR1*) and human homolog of the Drosophila dishevelled gene (*DVL1*) were found by pairwise comparison analysis to be differentially expressed between lobular and ductal carcinomas. Immunohistochemistry confirmed higher expression of DVL1 and EMP1 in lobular carcinomas and of DDR1 in ductal carcinomas (*p *< 0.0001) (Table 7 and Figure 5). Of the special type carcinomas included on TMA slides, a papillary and two medullary carcinomas were positive for basal cytokeratins, one out of three medullary carcinomas was positive for EMP1 and E-cadherin, a ductal-lobular carcinoma was positive for DDR1, all other special type carcinomas were negative for these markers, and finally none were positive for DVL1 (data not shown).

### Validation of asporin expression by *in situ *hybridization

**Figure 6:**
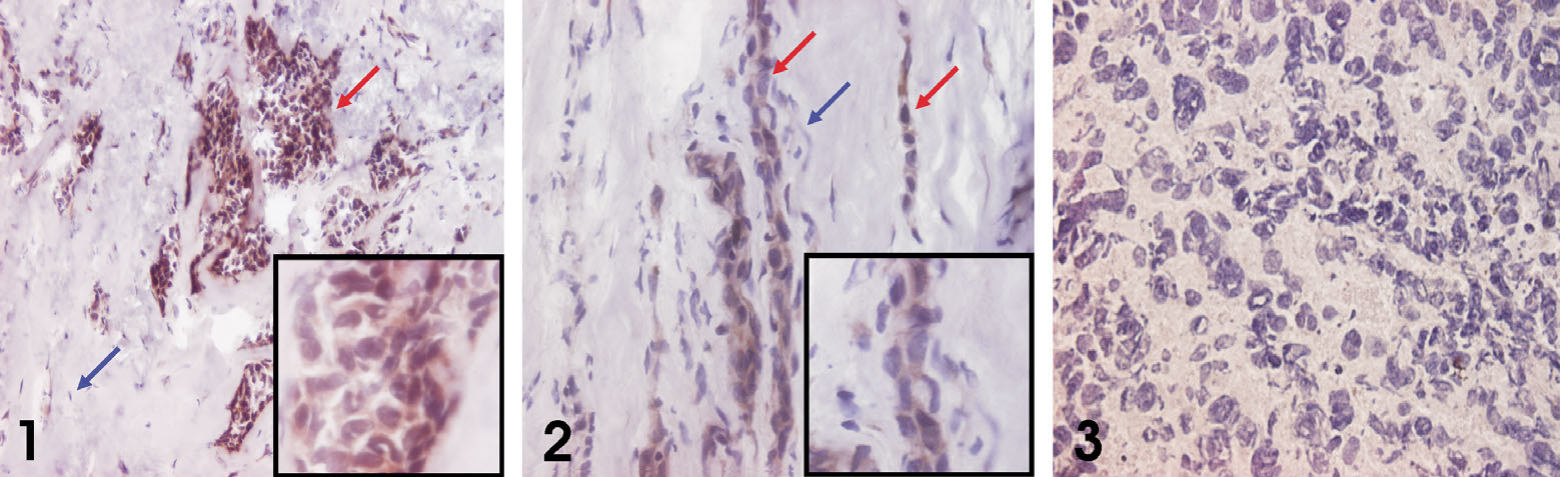
**Asporin mRNA detection by chromogenic *in situ *hybridization**. 1–2. Various magnifications (×100, ×400, ×400) of lobular carcinoma; red arrows, lobular tumor cells are positive; blue arrows, stromal cells are negative; 3. Ductal carcinoma is negative (×200).

Combined RP and pairwise comparison showed that *ASPN *was one of the most upregulated genes in lobular carcinomas, when compared with normal ductal (fold change 23.3) and lobular (fold change 22.1) cells as well as with ductal carcinomas (fold change 3.9). None of the available antibodies against asporin worked on paraffin sections, therefore we utilised chromogenic *in situ *hybridization to detect asporin mRNA in frozen sections. All five lobular carcinomas appeared positive, whereas five cases of ductal carcinomas were negative or weakly and focally positive which is in agreement with the microarray data (Figure 6).

### Validation of *CTHRC1, ASPN *and *COL3A1 *by PCR

**Figure 7:**
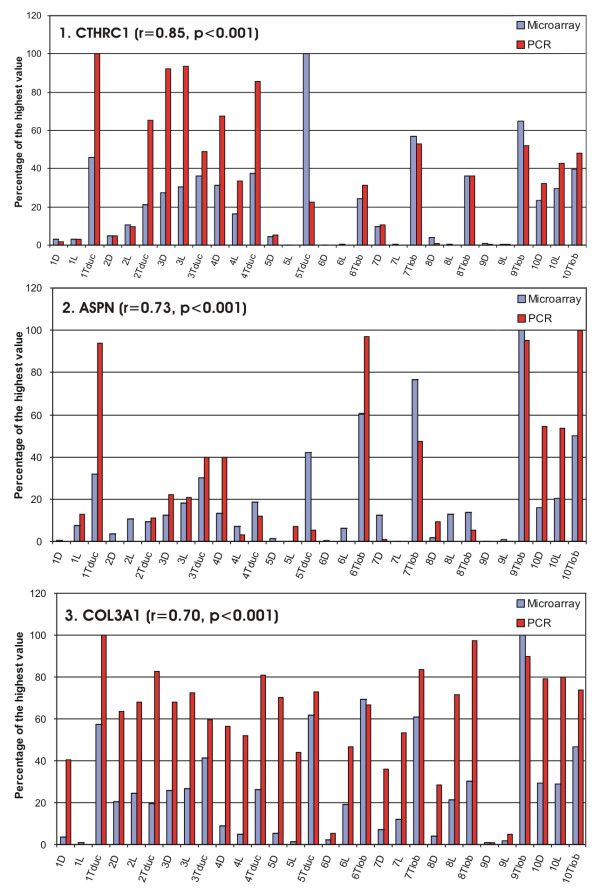
**PCR validation of microarray results for *CTHRC1 *(1), *ASPN *(2), and *COL3A1 *(3)**. Fluorescence signals from Affymetrix probe sets (225681_at, 219087_at and 211161_s_at, respectively) and optical density of PCR bands (OD × cm) were transformed to a percentage of the highest value. 1–10, case numbers; Tduc, ductal tumor; Tlob, lobular tumor; D, normal ductal cells; L, normal lobular cells.

Besides asporin, *CTHRC1 *was also upregulated in lobular cancer cells, when compared with normal ductal cells (fold change 48.4) and lobular cells (fold change 18.5), and *COL3A1 *was upregulated in both IDC and ILC cells, when compared with normal ductal cells (fold change 3.3 and 14.9, respectively) and lobular cells (fold change 4.1 and 9.8, respectively). As both RNA and cDNA were fully utilized for amplification, we used PCR amplification products (see Methods) for validation by another PCR with specific primers. The results of semi-quantitative PCR correlated with the microarray data. Spearman coefficients were r = 0.85, r = 0.73 and r = 0.70 for *CTHRC1*, *ASPN *and *COL3A1*, respectively (all significant at *p *< 0.0001) (Figure 7).

## Discussion

We examined 30 samples (microdissected tumor and normal ductal and lobular cells) from postmenopausal patients with lobular and ductal invasive breast carcinomas using Affymetrix arrays. Genes differentially expressed between the normal ductal and lobular cell types, which are less likely to be affected by fluctuating levels of female hormones as they were derived from postmenopausal women, are involved in ion transport and protein kinase cascade, protein biosynthesis and modification, proteolysis and metabolism, regulation of transcription and cell growth. However, hierarchical clustering of normal cell types based on the 82 probe sets identified by both RP and pairwise comparison showed that the gene expression profiles of normal ductal and lobular cells taken from the same patient are more similar to each other than ductal cells or lobular cells from different patients, and thus could not be well separated from each other. This may suggest expected similarities between expression signatures in normal epithelial (ductal and lobular) cells of the mammary gland. Since both IDC and ILC are believed to start in the terminal duct lobular unit (TDLU) of the breast [1,2], and these normal cell populations showed similar gene expression profiles, it is likely that the different morphological appearances of the two tumor types are mediated by differences in their mechanisms of carcinogenesis.

Combined pairwise comparison and RP analysis revealed that ductal and lobular carcinomas have a number of genes in common, however, they can be discriminated both at the gene and protein levels in our study. cDNA microarrays have previously been used to distinguish between IDC and ILC [38,39]. Unsupervised clustering of tumors failed to separate the two subtypes. There were 8 genes identified by MaxT permutation analysis using t tests, significance analysis for microarrays (SAM) and prediction analysis for microarrays (PAM) (E-cadherin, survivin, cathepsin B, TPI1, SPRY1, SCYA14, TFAP2B, and thrombospondin 4), and an additional 3 were identified by SAM and PAM (osteopontin, HLA-G, and CHC1) [38]. It has also been found that over half of ILCs differed from IDCs in global transcription programs, whereas the remaining ILCs closely resembled IDCs. Fifty two percent of the ILCs ("typical" ILCs) clustered together and displayed different gene expression profiles from the IDCs, whereas the other ILCs ("ductal-like" ILCs) were distributed between different IDC subtypes. Many of the differentially expressed genes encode for proteins involved in cell adhesion/motility, lipid/fatty acid transport and metabolism, immune/defense response, and electron transport. Many genes distinguishing between typical and ductal-like ILCs are involved in regulation of cell growth and immune response [39]. However, these two previous studies examined whole tumor tissues without microdissection, suggesting that expression of a number of genes could be related not only to tumor cells but also to other components of mammary tissue such as stromal, adipose, endothelial etc. Our study is the first full genome analysis of microdissected ductal and lobular tumor and normal cells reporting both normal mammary epithelium- and cancer-specific genes expression profiles.

Importantly, *CDH1 *(E-cadherin gene) was downregulated in our lobular carcinomas, and immunohistochemistry confirmed this loss at the protein level within tumors. E-cadherin is considered to be the most important cell adhesion molecule in the mammary gland. It acts as a tumor suppressor inhibiting invasion and metastasis. Mutations of this gene are correlated with gastric, breast, colorectal, thyroid and ovarian cancer. During tumor progression, E-cadherin can be functionally inactivated or silenced by different mechanisms such as post-translational control, somatic mutations, downregulation of gene expression through promoter hypermethylation, histone deacetylation, and transcriptional repression [52,53]. The latter induces cellular responses leading to the conversion of epithelial cells into invasive mesenchymal-like cells with increased motility and invasiveness, and this process is called an epithelial-mesenchymal transition (EMT) [52]. To date, it is believed that lost, non-polar or cytoplasmic expression of E-cadherin protein and/or transcriptional repression of its mRNA are hallmarks of EMT in cancer progression [53-55]. It has also been shown that several proteins such as fibronectin and integrin αvβ6 [54], Ets, TGFβ, FGF-1,-2,-8, α-SMA, collagen type I, III and thrombospondins increase in abundance during EMT [56], conversely, amongst proteins that decrease in abundance are E-cadherin and cytokeratins [54]. According to our results, collagen type I and III, fibronectin and Ets domain transcription factor are upregulated and cytokeratins are downregulated in both tumor cell types. The expression of collagens and other mesenchyme-associated genes in microdissected breast cancer cells was also confirmed by Nishidate and co-workers [57]. In addition, pairwise comparisons revealed that thrombospondin 4 was upregulated only in lobular cancer cells, which agrees with the literature [38]. Thus we propose that the EMT plays a role in both tumor types but appears to be more important in lobular carcinomas. The EMT phenomenon seems to be promising because multiple molecules involved in EMT, such as receptor- and SRC-family tyrosine kinases, RAS and other small GTPases, can be envisioned as targets for anti-EMT therapy [58].

The tissue microenvironment, including the ECM-cell and cell-cell interactions, plays an important role in both normal mammary gland development and cancer. Neoplastic transformation of cells dramatically alters the synthesis of proteoglycans and other ECM proteins both in tumor and the surrounding matrix [59]. This can stimulate the growth and spread of tumor cells by decreasing the adhesive functions of the ECM [60]. Both tumor types examined show upregulated genes involved in tumor-ECM interactions, cell adhesion and migration processes including metastasis. Expression of majority of the proteins encoded by these genes is related to TGFβ or Wnt signaling, and both the TGFβ and Wnt pathways may affect ECM.

Asporin is a cartilage extracellular protein that has been reported to be associated with knee and hip osteoarthritis. This leucine-rich repeat protein was shown to interact with and inhibit TGFβ signaling which is thought to lead to insufficient quantities of aggrecan and type II collagen in osteoarthritis [61,62]. Asporin was more upregulated in our lobular carcinomas when compared with ductal tumors as well as with normal cell types. Overexpression of asporin mRNA in lobular carcinomas was then confirmed by chromogenic *in situ *hybridization and PCR. In support of our findings, upregulation of this gene has also been described in microdissected androgen-independent prostate cancer cells using Affymetrix Human Genome U133A GeneChips [63]. The authors did not discuss it and importantly, asporin has not been related to carcinogenesis to date.

We have found another candidate gene, collagen triple helix repeat containing 1 (*CTHRC1*), which was upregulated in ILC in comparison with normal cells, and its expression was also validated by PCR. Aberrant expression of *CTHRC1 *has recently been reported in human solid tumors, including cancers of the gastrointestinal tract, lung, breast, thyroid, ovarian, cervix, liver, and the pancreas. It is associated with cancer tissue invasion and metastasis and potentially plays important functional roles in cancer progression, perhaps by increasing cancer cell migration [64]. TGFβ upregulates CTHRC1, versican, ADAM12, and downregulates SFRP1 and E-cadherin [65-67]. The loss of SFRP1 is known to be associated with breast cancer progression and poor prognosis in early stages [68], and a similar expression profile is seen in our study. *SFRP1 *is downregulated and versican is upregulated in both tumor types. Furthermore, pairwise comparison identified other overexpressed genes in lobular carcinoma such as *SFRP2 *and *ADAM12*. E-cadherin, which is downregulated in lobular cancer cells, can also be repressed by TGFβ-induced expression of transcription factor complexes [69]. TGFβ signaling inhibitors have been shown to prevent EMT, to inhibit mammary tumor viability and to block metastasis in various murine models [70]. According to these results, we propose that deregulated TGFβ signaling is likely to be more important in lobular carcinogenesis.

Wnt signaling molecules are also expressed in our samples in several comparisons. The Wnt proteins are small secreted glycoproteins which are involved in the self-renewal of stem cells and may be responsible for the maintenance of mature tissues [71]. On binding to Frizzled receptors, Wnts can activate canonical (β-catenin-dependent) and/or non-canonical (β-catenin-independent Wnt/planar cell polarity pathway and Wnt/Ca2+ pathway) Wnt signaling [72,73]. Downregulation of frizzled related proteins has been described in breast cancer [68]. Of these, *SFRP1 *is downregulated in both types of our tumor cells, and genes involved in calcium regulation pathway are not significantly changed. The promoter of fibronectin 1 contains LEF/TCR-binding sites, making it a direct target of canonical Wnt signaling [59,74]. Fibronectin 1 is also upregulated in both tumor types. However, there is a difference in Wnt signaling between ILC and IDC. *SULF1 *(sulfatase 1) is upregulated and *MMP7 *is downregulated only in IDCs. *MMP7 *is a confirmed Wnt target [75] and it has been shown to be activated in both canonical [76,77] and non-canonical Wnt signaling [78]. Binding Wnt ligands to frizzled receptors is regulated by the 6-O sulfation-desulfation of cell surface heparan sulfates (HSs) by sulfatase 1. Sulfated HSs bind to Wnt ligand with high affinity and inhibit Wnt signaling. Sulfatase 1 removes 0–6 sulfates from HSs and reduces their binding to Wnt ligands which in turn allows the formation of functional Wnt-Frizzled complexes and thus promotes Wnt signaling [79,80]. Frizzled-related proteins also have heparin-binding domains that promote the formation of Wnt-Frizzled complexes [81]. Although *SULF1 *is upregulated, *SFRP1 *and *MMP7 *are downregulated in ductal cancer cells, whereas *SFRP2 *and other Wnt molecules, such as *DVL1 *and *WISP1*, are upregulated in lobular cancer cells. This suggests that Wnt signaling is activated in ILC cells but not in IDC cells. Since mRNA level of β-catenin was not changed between normal and tumor cells, the expression of Wnt molecules appears to be β-catenin-independent, favoring the non-canonical Wnt signaling in ILC. There is evidence that Wnts acting through the non-canonical pathway can promote tumor progression [82,83] which may also be true in ILC.

In addition to Wnt molecules such as *DVL1*, pairwise comparison revealed that *EMP1*, gene encoding a tumor-associated membrane protein involved in cell-cell interactions and proliferation control [84], was upregulated, whereas *DDR1*, epithelial-specific receptor kinase capable of binding Wnt5 and regulating the adhesion of mammary cells [85,86], was downregulated in ILCs. Immunohistochemistry has also confirmed the same differential expression of these three proteins in IDC and ILC on tissue microarrays. Thus all the evidence suggests that the two tumor types can be distinguished both at the gene and protein levels. Specific changes in gene and protein expression are likely to reflect the differences in mechanisms of carcinogenesis as well as the specific histological and clinical characteristics of these tumors derived from the same anatomical compartment, TDLU.

## Conclusion

Microdissection of normal and tumor cell types from the breast and full genome expression analysis by Affymetrix arrays allowed us to provide novel data on breast cancer. Invasive lobular and ductal breast carcinomas can be differentiated both at the gene and protein levels. Despite analyzing only thirty samples from ten patients, the results are in good accordance with previous literature [14,38,39,57,64]. Our data provide evidence for deregulated TGFβ and Wnt signaling accompanied by the overexpression of mesenchyme-associated genes like the collagens, asporin and others which might be occurring in conjunction with an altered EMT. We propose that deregulated TGFβ signaling and EMT phenomenon are involved in both tumor types, but they seem to be more important in lobular carcinomas which is in concordance with the loss of E-cadherin expression and their distinct morphology from ductal tumors. In this study we report two candidate genes, asporin (*ASPN*) and collagen triple helix repeat containing 1 (*CTHRC1*), which might be significant in mammary gland carcinogenesis and may also be important either in cancer diagnosis or therapy. Besides E-cadherin, the proteins validated on tissue microarrays by immunohistochemistry (EMP1, DVL1, DDR1) may represent novel tissue markers helpful in the differentiation of ductal and lobular cancers. Further studies with larger sets of patients are needed to verify the gene expression profiles of various histological types of breast cancer in order to determine molecular subclassifications, prognosis, and the optimum treatment strategies.

## Abbreviations

ADAM12 = ADAM metallopeptidase domain 12; ASPN = Asporin; CK = Cytokeratin; CTHRC1 = Collagen triple helix repeat containing 1; DAVID = Database for Annotation, Visualization and Integrated Discovery; DDR1 = Discoidin domain receptor 1; DVL1 = Human homolog of the Drosophila dishevelled gene; ECM = Extracellular matrix; EMP1 = Epithelial membrane antigen 1; EMT = Epithelial-mesenchymal transition; ER = Estrogen receptor; GCOS = GeneChip Operating Software; IDC = Invasive ductal carcinoma; ILC = Invasive lobular carcinoma; ISH = *In situ *hybridization; IVT = *In vitro *transcription; PCR = Polymerase chain reaction; PgR = Progesterone receptor; RMA = Robust multiarray analysis; RNA = Ribonucleic acid; RP = Rank products analysis; SFRP1 = Secreted frizzled-related protein 1; TDLU = Terminal duct lobular unit; TGFβ = Transforming growth factor β ; TMA = Tissue microarray; WISP1 = WNT1 inducible signaling pathway protein 1.

## Competing interests

The author(s) declare that they have no competing interests.

## Authors' contributions

GT and JB contributed equally to this work. GT participated in collecting surgical specimens, cutting and evaluation of frozen sections, laser microdissection, immunohistochemistry, ISH, analysis of microarray results and drafting the manuscript. JB participated in the design of the study, collecting surgical specimens, RNA isolation, amplification and labeling, analysis of microarray results, ISH and PCR validation, drafting the manuscript. KB carried out the hybridization and scanning of the arrays and proofreading of the manuscript. WW performed data analysis and participated in drafting the manuscript. MD and MH were involved in laser microdissection. JEparticipated in collection and evaluation of frozen sections. JK participated in obtaining surgical specimens. EF and JS participated in tissue microarray construction. JS was involved in RNA isolation and PCR validation. PM participated in analysis of microarray results. ZK conceived of the study, participated in its design and coordination, as well as in collecting surgical specimens, evaluation of frozen sections and drafting the manuscript. All authors read and approved the final manuscript.

## Pre-publication history

The pre-publication history for this paper can be accessed here:



## Supplementary Material

Additional File 1 Supplementary tables. The data provided represent supplementary tables listing all differentially expressed probe sets found by both rank products and pairwise analysis between normal ductal and normal lobular cells, ductal carcinoma and normal ductal cells, ductal carcinoma and normal lobular cells, ductal carcinoma and lobular carcinoma cells, lobular carcinoma and normal ductal cells, and lobular carcinoma and normal lobular cells. Click here for file

## Figures and Tables

**Table 1:** Clinical and histopathological characteristics of breast cancer patients involved in microarray analysis

**No**	**Diagnosis**	**Grade**^**1**^	**AC**	**LN**	**ER**^**3**^	**PgR**^**3**^	**HER-2**	**Bcl-2**	**E-CD**
1	IDC	G3	IDH	N_1_	0	0	0	80%	>90%
2	IDC	G2	none	N_1_	40%	0	0	80%	>90%
3	IDC	G2	none	N_0_	10%	2%	0	70%	>90%
4	IDC	G3	none	N_0_	90%	50%	2+ ^2^	100%	>90%
5	IDC	G2	DCIS	N_0_	90%	1%	0	95%	>90%
6	ILC	G2	LCIS	N_0_	0	0	0	30%	0
7	ILC	G1	none	N_0_	80%	80%	0	100%	0
8	ILC	G1	none	N_1_	50%	0	0	80%	0
9	ILC	G2	LCIS	N_1_	0	0	0	<5%	0
10	ILC	G1	none	N_0_	50%	70%	0	80%	0

^1^Tumors were graded using the Nottingham combined histologic grading system.

^2^No gene amplification was detected by fluorescent *in situ *hybridization.

^3^A complete H-score was calculated by summing the products of the percentage cells stained at a given staining intensity (0–100) and the staining intensity (0–3).

AC, accompanying changes; DCIS, ductal carcinoma *in situ*; E-CD, E-cadherin; ER, estrogen receptor; IDC, invasive ductal carcinoma, IDH, intraductal hyperplasia; ILC, invasive lobular carcinoma; LCIS, lobular carcinoma *in situ*; LN, lymph node; PgR, progesterone receptor.

**Table 2:** Genes differentially expressed between normal lobular versus normal ductal cells by both rank products and pairwise analysis

**Probe Set**^**1**^	**Gene Symbol**	**Gene Title**	**Rank products**	**Pairwise comparison**
			
			**Fold-change**	**Incr/Decr**
***Regulation of transcription***				
229431_at	*RFXAP*↓	Regulatory factor X-associated protein	-3.1	1/6
229732_at	*HSZFP36*↓	ZFP-36 for a zinc finger protein	-3.0	0/6
207117_at	*H-plk*↑	Krueppel-related zinc finger protein	2.4	6/1
***Protein biosynthesis and modification***				
214130_s_at	*PDE4DIP*↓	Phosphodiesterase 4D interacting protein (myomegalin)	-3.2	1/6
232752_at	*--- (LOXL1*) ↓	CDNA FLJ10302 fis, clone NT2RM2000042 (lysyl oxidase-like 1)	-1.4	1/6
***Proteolysis and metabolism***				
49452_at	*ACACB*↓	Acetyl-Coenzyme A carboxylase beta	-18	0/8
220419_s_at	*USP25*↓	Ubiquitin specific peptidase 25	-2.9	1/6
220177_s_at	*TMPRSS3*↓	Transmembrane protease, serine 3	-2.8	1/6
***Ion transport***				
201735_s_at	*CLCN3*↓	Chloride channel 3	-2.8	2/6
205044_at	*GABRP*↓	Gamma-aminobutyric acid (GABA) A receptor, pi	-1.8	1/6
***Actin binding***				
212650_at	*EHBP1*↑	EH domain binding protein 1	2.6	6/0
***GTPase activity***				
202975_s_at	*RHOBTB3*↑	Rho-related BTB domain containing 3	2.5	7/1
203264_s_at	*ARHGEF9*↑	Cdc42 guanine nucleotide exchange factor (GEF) 9	2.6	6/0
***Regulation of cell development, activation, growth and motility***				
209890_at	*TSPAN5*↑	Tetraspanin 5	2.6	7/0
***Small leucine-rich proteoglycan (slrp) family***				
211896_s_at	*DCN*↑	Decorin	2.0	7/1
***Protein kinase cascade***				
211081_s_at	*MAP4K5*↓	Mitogen-activated protein kinase kinase kinase kinase 5	-2.3	1/7
***Other***				
206157_at	*PTX3*↓	Pentraxin-related gene, rapidly induced by IL-1 beta	-2.8	2/7
218431_at	*C14orf133*↓	Chromosome 14 open reading frame 133	-2.7	0/7
218514_at	*FLJ10587*↓	Hypothetical protein FLJ10587	-2.6	1/7
226760_at	*LOC203411*↑	Hypothetical protein LOC203411	3.0	6/2

↑ – upregulated in normal lobular cells; ↓ – downregulated in normal lobular cells

^1^Affymetrix probe set number; If there was more than one probe set for the particular gene found among top 20 changed genes (according to the fold change), the probe set with the highest fold change is used and number of other probe set is indicated.

**Table 3:** Genes differentially expressed between tumor versus normal cells by both rank products and pairwise analysis

**Probe Set**^**1**^	**Gene Symbol**	**Gene Title**	**Fold-change**			
			
			**Tduc**		**Tlob**	
**Lobular carcinoma vs normal cells** **(↑ – upregulated in Tlob, ↓ – downregulated in Tlob)**			**L**	**D**	**L**	**D**

***Extracellular matrix-receptor interaction and focal adhesion (epithelial-mesenchymal transition***)						
227314_at	*ITGA2*↓	Integrin, alpha 2	n	n	-4.7	-6.2
201438_at	*COL6A3*↑	Collagen, type VI, alpha 3	n	n	4.5	7.7
203083_at	*THBS2*↑	Thrombospondin 2	n	n	6.5	6.5
205713_s_at	*COMP*↑	Cartilage oligomeric matrix protein	n	n	6.7	8.1
226237_at	*COL8A1*↑	Collagen, type VIII, alpha 1	n	n	8.4	5.9
225681_at	*CTHRC1*↑	Collagen triple helix repeat containing 1	n	n	18.5	48.4
219087_at	*ASPN*↑	Asporin	n	n	23.3	22.1
***WNT signalling***						
223121_s_at 223122_s_at	*SFRP2*↑	Secreted frizzled-related protein 2	n	n	5.8 3.9	7.4 7.1
229802_at	*WISP1*↑	WNT1 inducible signaling pathway protein 1	n	n	9.1	12.4
***Actin, calcium and metal ion binding***						
204455_at	*DST*↓	Dystonin	n	n	-22.2	-18.3
214087_s_at	*MYBPC1*↓	Myosin binding protein C, slow type	n	n	-10.1	-12.2
209301_at	*CA2*↓	Carbonic anhydrase II	n	n	-9.2	-8.7
206509_at	*PIP*↓	Prolactin-induced protein	n	n	-7.0	-6.3
201131_s_at	*CDH1*↓	Cadherin 1, type 1, E-cadherin	n	n	-6.2	-7.2
226777_at	*ADAM12*↑	ADAM metallopeptidase domain 12	n	n	5.7	5.3
213905_x_at	*BGN/SDCCAG33*↑	Biglycan/serologically defined colon cancer antigen 33	n	n	8.3	7.2
***Other***						
227742_at	*CLIC6*↓	Chloride intracellular channel 6	n	n	-9.3	-8.2
225817_at	*CGNL1*↓	Cingulin-like 1	n	n	-5.9	-7.8
219850_s_at	*EHF*↓	Ets homologous factor	n	n	-4.1	-6.3
213975_s_at	*LYZ/ILRB1*↑	Lysozyme	n	n	10.8	7.3

**Ductal carcinoma vs normal cells** **(↓ – upregulated in Tduc, ↓ – downregulated in Tduc)**						

***Regulation of transcription***						
202672_s_at	*ATF3*↓	Activating transcription factor 3	-4.5	-7.7	n	n
226147_s_at	*PIGR*↓	Polymeric immunoglobulin receptor	-4.8	-10	n	n
***Cytokine and growth factor activity***						
209466_x_at 211737_x_at	*PTN*↓	Pleiotrophin	-5.8 -7.1	-8.3 -12.5	n	n
823_at	*CX3CL1*↓	Chemokine (C-X3-C motif) ligand 1	-3.6	-8.3	n	n
***Ion transport and metabolism***						
203296_s_at	*ATP1A2*↓	ATPase, Na+/K+ transporting, alpha 2	-8.2	-9.6	n	n
204259_at	*MMP7*↓	Matrix metallopeptidase 7	-4.5	-7.2	n	n
212353_at	*SULF1*↓	Sulfatase 1	4.6	4.0	n	n
***Other***						
1553602_at	*LOC118430*↓	Small breast epithelial mucin	-7.1	-10.4	n	n
223623_at	*ECRG4*↓	Esophageal cancer related gene 4 protein	-6.6	-5.6	n	n
206548_at	*FLJ23556*↓	Hypothetical protein FLJ23556	-6.3	-11.2	n	n
218585_s_at	*DTL*↑	Denticleless homolog (Drosophila)	3.6	4.8	n	n

**Tumor vs normal cells** **(↑ – upregulated in tumor, ↓ – downregulated in tumor)**						

***Extracellular matrix-receptor interaction and focal adhesion (epithelial-mesenchymal transition***)						
224823_at	*MYLK*↓	Myosin, light polypeptide kinase	-3.5	-3.4	-5.9	-5.4
204620_s_at 221731_x_at	*CSPG2*↑	Chondroitin sulfate proteoglycan 2 (versican)	3.0 3.5	4.9 5.3	4.5 4.0	5.8 6.1
221729_at	*COL5A2*↑	Collagen, type V, alpha 2	3.5	4.4	9.3	11.3
211161_s_at	*COL3A1*↑	Collagen, type III, alpha 1	4.1	3.3	9.8	14.9
212464_s_at	*FN1*↑	Fibronectin 1	5.2	5.0	8.0	10.5
202311_s_at 202310_s_at	*COL1A1*↑	Collagen, type I, alpha 1	5.6 5.1	6.0 5.1	12.7 10.4	13.0 10.8
37892_at	*COL11A1*↑	Collagen, type XI, alpha 1	6.7	7.8	6.6	5.9
202404_s_at	*COL1A2*↑	Collagen, type I, alpha 2	6.8	10.1	30.5	18.3
***Ion and electron transport***						
205044_at	*GABRP*↓	Gamma-aminobutyric acid (GABA) A receptor, pi	-4.2	-8.7	-6.2	-8.6
220230_s_at	*CYB5R2*↓	Cytochrome b5 reductase 2	-3.6	-3.8	-4.3	-4.2
***Regulation of transcription***						
220625_s_at	*ELF5*↓	E74-like factor 5 (ets domain transcription factor)	-4.0	-4.8	-5.9	-8.4
209292_at	*ID4*↓	Inhibitor of DNA binding 4	-2.9	-5.1	-10.8	-4.5
***Actin and actin-binding proteins***						
203951_at	*CNN1*↓	Calponin 1, basic, smooth muscle	-6.7	-6.6	-6.9	-7.3
243140_at	*ACTA2*↓	Actin, alpha 2, smooth muscle, aorta	-4.0	-4.1	-8.7	-4.0
***WNT signaling***						
202037_s_at	*SFRP1*↓	Secreted frizzled-related protein 1	-7.1	-8.8	-8.5	-7.3
***Intermediate filament proteins***						
209351_at	*KRT14*↓	Keratin 14	-22.9	-31.0	-21.9	-24.0
205157_s_at	*KRT17*↓	Keratin 17	-17.7	-21.4	-9.9	-12.6
212730_at	*DMN*↓	Desmuslin	-10.0	-10.4	-7.2	-7.5
201820_at	*KRT5*↓	Keratin 5	-8.9	-12.7	-11.3	-12.1
***Other***						
229947_at	*PI15*↓	Peptidase inhibitor 15	-6.5	-7.5	-18.5	-15.0
205380_at	*PDZK1*↓	PDZ domain containing 1	-4.5	-5.8	-4.2	-6.4
213909_at	*LRRC15*↑	Leucine rich repeat containing 15	6.2	5.7	7.3	11.8

Tduc – ductal tumor; Tlob – lobular tumor; D – normal ductal cells; L – normal lobular cells; n – not differentially expressed among top 20 genes.

^1^Affymetrix probe set number; If there was more than one probe set for the particular gene found among top 20 changed genes (according to the fold change), the probe set with the highest fold change is used and number of other probe set is indicated.

**Table 4:** Genes differentially expressed in lobular versus ductal invasive carcinomas by rank products and/or pairwise analysis

**Probe Set**^**1**^	**Gene Symbol**	**Gene Title**	**Rank products**	**Pairwise comparison**
			
			**Fold-change**	**Incr/Decr**
***Cell adhesion***				
201131_s_at	*CDH1*↓	Cadherin 1, type 1, E-cadherin (epithelial)	-6.0	3/19
207169_x_a	*DDR1*↓	Discoidin domain receptor family, member 1	n	5/19
204776_at	*THBS4*↑	Thrombospondin 4	n	19/2
219087_at	*ASPN*↑	Asporin (LRR class 1)	3.9	23/0
***Wnt signalling***				
223168_at	*RHOU*↓	RAS homolog gene family, member U	n	2/20
203230_at	*DVL1*↑	Dishevelled, dsh homolog 1 (Drosophila)	n	20/0
***Protein ubiquitination***				
215392_at	*USP3*↓	Ubiquitin specific peptidase 3	-5.0	1/21
218247_s_at	*RKHD2*↓	Ring finger and KH domain containing 2	-4.6	0/19
1569472_s_at	*TTC3*↓	Tetratricopeptide repeat domain 3	-4.5	0/23
***Regulation of transcription***				
226115_at	*AHCTF1*↓	AT hook containing transcription factor 1	-4.0	0/22
201587_s_at	*IRAK1*↓	Interleukin-1 receptor-associated kinase 1	n	2/19
202599_s_at	*NRIP1*↓	Nuclear receptor interacting protein 1	n	0/19
201773_at	*ADNP*↓	Activity-dependent neuroprotector	n	0/20
203637_s_at	*MID1*↑	Midline 1 (Opitz/BBB syndrome)	4.0	20/0
235444_at	*FOXP1*↑	Forkhead box P1	n	19/4
***Electron and protein transport***				
204351_at	*S100P*↓	S100 calcium binding protein P	-5.3	0/19
225177_at	*RAB11FIP1*↓	RAB11 family interacting protein 1 (class I)	-3.5	2/21
204041_at	*MAOB*↑	Monoamine oxidase B	3.5	20/4
212902_at	*SEC24A*↑	SEC24 related gene family, member A	3.5	19/2
***Cell cycle regulation, cell proliferation and signaling***				
204170_s_at	*CKS2*↓	CDC28 protein kinase regulatory subunit 2	-4.8	2/20
207414_s_at	*PCSK6*↓	Proprotein convertase subtilisin/kexin type 6	n	4/19
209678_s_at	*PRKCI*↓	Protein kinase C, iota	n	0/22
209817_at	*PPP3CB*↓	Protein phosphatase 3, catalytic subunit, beta isoform (calcineurin A beta)	n	0/24
201324_at	*EMP1*↑	Epithelial membrane protein 1	n	21/0
***Metabolism and enzyme activity***				
205509_at	*CPB1*↓	Carboxypeptidase B1 (tissue)	-11.1	0/19
223746_at	*STK4*↓	Serine/threonine kinase 4	-4.5	2/20
225491_at	*SLC1A2*↓	Solute carrier family 1 (glial high affinity glutamate transporter), member 2	-4.1	0/20
211379_x_at	*B3GALT3*↓	UDP-Gal:betaGlcNAc beta 1,3-galactosyltransferase, polypeptide 3	-4.0	0/20
219073_s_at	*OSBPL10*↓	Oxysterol binding protein-like 10	-4.0	1/21
229366_at	*CRBN*↓	Cereblon	-3.6	0/20
226350_at	*CHML*↓	Choroideremia-like (Rab escort protein 2)	n	0/24
217717_s_at	*YWHAB*↓	Tyrosine 3-monooxygenase/tryptophan 5-mono-oxygenase activation protein, beta polypeptide	n	1/20
49452_at	*ACACB*↑	Acetyl-Coenzyme A carboxylase beta	n	19/0
220770_s_at	*LOC63920*↑	Transposon-derived Buster3 transposase-like	5.3	24/0
***Actin and cytoskeletal protein binding***				
211776_s_at	*EPB41L3*↓	Erythrocyte membrane protein band 4.1-like 3	-5.3	0/21
201341_at	*ENC1*↓	Ectodermal-neural cortex (with BTB-like domain)	n	19/3
***Nuclear components***				
218585_s_at	*DTL*↓	Denticleless homolog (Drosophila)	-4.6	1/19
225706_at	*GLCCI1*↓	Glucocorticoid induced transcript 1	-4.5	0/25
241863_x_at	*TTC14*↓	Tetratricopeptide repeat domain 14	-4.4	1/20
228069_at	*FAM54A*↓	Family with sequence similarity 54, member A	-3.8	0/20
208576_s_at	*HIST1H3B*↓	Histone 1, H3b	-3.3	1/19
***Other***				
1568838_at	--- ↓	Full-length cDNA clone CS0DF033YE17 of Fetal brain of Homo sapiens (human)	-5.0	1/20
226067_at	*C20orf114*↓	Chromosome 20 open reading frame 114	-4.9	0/19
1553979_at	--- ↓	Homo sapiens, clone IMAGE:3906992, mRNA	-4.1	0/19
230534_at	*MGC15634*↓	Hypothetical protein MGC15634	-3.6	2/20
226671_at	--- ↓	CDNA clone IMAGE:4797120	-3.8	2/20
219768_at	*VTCN1*↑	V-set domain containing T cell activation inhibitor 1	7.8	21/2
214657_s_at	*TncRNA*↑	Trophoblast-derived noncoding RNA	4.5	21/0
213004_at	*ANGPTL2*↑	Angiopoietin-like 2	n	19/4

↑ – upregulated in lobular carcinoma; ↓ – downregulated in lobular carcinoma; n – gene not found by rank products at the level of 10% of false positives, Incr/decr – increase or decrease in lobular versus ductal carcinoma by pairwise comparison.

^1^All 32 probe sets identified by combined pairwise comparison and rank products are included, also a number of genes found by pairwise comparison were validated by immunohistochemistry (DVL1, DDR1, EMP1), therefore, functional categories also include named genes identified by pairwise analysis.

**Table 5:** Clinical and histopathological characteristics of TMA cases

**Features**	**IDC**	**ILC**
G1	6 (7.5%)	15 (51.7%)
G2	57 (71.3%)	11 (37.9%)
G3	17 (21.2%)	3 (10.3%)
ER+	57 (71.3%)	19 (65.5%)
PgR+	50 (62.5%)	17 (58.6%)
HER-2+	12 (15%)	1 (3.4%)
Bcl-2+	13 (16.3%)	4 (13.8%)
N_0_	56 (70%)	21 (72.4%)
N_1_	16 (20%)	6 (20.7%)
N_2_	8 (10%)	2 (6.9%)
Total number of cases	80	29

ER, estrogen receptor; G1-3, grade 1–3; IDC, invasive ductal carcinoma; ILC, invasive lobular carcinoma; N_0–1_, lymph node status; PgR, progesterone receptor; TMA, tissue microarray.

**Table 6:** Immunohistochemical staining of cytokeratins in tumor and normal tissues

**Antibodies**	**Tumors**		**Normal tissues (n = 22)**	**Fisher's *p *value**
Cytokeratin 5/6	**Ductal carcinomas (n = 80)**	9 (11.2%)	22 (100%)	<0.0001
	**Lobular carcinomas (n = 29)**	3 (10.3%)		<0.0001
Cytokeratin 17	**Ductal carcinomas (n = 80)**	8 (10%)	22 (100%)	<0.0001
	**Lobular carcinomas (n = 29)**	2 (6.9%)		<0.0001

**Table 7:** Immunohistochemistry differentiates ductal and lobular carcinomas

**Antibodies**	**Ductal carcinomas (n = 80)**	**Lobular carcinomas (n = 29)**	**Fisher's *p *value**
E-cadherin	68 (85%)	2 (6.7%)	<0.0001
DVL1	20 (25%)	28 (96.5%)	<0.0001
EMP1	13 (16.3%)	27 (93.1%)	<0.0001
DDR1	77 (96.2%)	4 (13.8%)	<0.0001
