# Supplementary material for: Novel markers for differentiation of lobular and ductal invasive breast carcinomas by laser microdissection and microarray analysis
Source: BMC Cancer. 2007 Mar 27;7:55. doi: 10.1186/1471-2407-7-55 (PMC1852112; doi:10.1186/1471-2407-7-55)
Supplement: Additional File 1 — Supplementary tables. The data provided represent supplementary tables listing all differentially expressed probe sets found by both rank products and pairwise analysis between normal ductal and normal lobular cells, ductal carcinoma and normal ductal cells, ductal carcinoma and normal lobular cells, ductal carcinoma and lobular carcinoma cells, lobular carcinoma and normal ductal cells, and lobular carcinoma and normal lobular cells. [file 1471-2407-7-55-S1.doc]

**Supplementary tables**

| **Comparison** | **Number of differentially expressed probe sets (named genes) by:** | | |
| --- | --- | --- | --- |
|  | **pairwise analysis** | **rank products** | **both methods** |
| D vs L | 367(327) | 501(427) | 82(78) |
| Tduc vs D | 1055(888) | 604(462) | 326(257) |
| Tduc vs L | 792(686) | 347(279) | 171(147) |
| Tduc vs Tlob | 208(183) | 117(102) | 32(28) |
| Tlob vs D | 1022(864) | 350(269) | 201(162) |
| Tlob vs L | 983(830) | 344(279) | 208(172) |

**Pairwise analysis:**

Count of changes more than 4 (out of 5) was used for comparisons between tumour and normal cells.

Count of changes more than 6 (out of 10) was used for comparisons of normal cells in between.

Count of changes more than 19 (out of 25 inter-patient comparisons - every ductal carcinoma against every lobular carcinoma) was used for comparisons of tumour cells in between.

**Rank products analysis:**

FC, fold change for the specified comparison; pfp, percentage of false positivities

Supplementary Table 1. Differentially expressed probe sets between normal ductal (D) and normal lobular (L) cells found by both rank products and pairwise analysis.

| Probe Set | Accession | Gene Symbol | FC:(D vs L) | pfp | Increase | Decrease |
| --- | --- | --- | --- | --- | --- | --- |
| 214130_s_at | AI821791 | PDE4DIP | 3,234153 | 0,005 | 6 | 1 |
| 229431_at | AI742868 | RFXAP | 3,07787 | 0,0044 | 6 | 1 |
| 229732_at | AI417785 | HSZFP36 | 3,027551 | 0,0067 | 6 | 0 |
| 220419_s_at | NM_013396 | USP25 | 2,857959 | 0,0145 | 6 | 1 |
| 206157_at | NM_002852 | PTX3 | 2,828054 | 0,01 | 7 | 2 |
| 201735_s_at | NM_001829 | CLCN3 | 2,826456 | 0,0073 | 6 | 2 |
| 220177_s_at | NM_024022 | TMPRSS3 | 2,806624 | 0,0045 | 6 | 1 |
| 218514_at | NM_018149 | FLJ10587 | 2,759382 | 0,0144 | 7 | 1 |
| 211081_s_at | Z25426 | MAP4K5 | 2,720348 | 0,016 | 7 | 1 |
| 218431_at | NM_022067 | C14orf133 | 2,688895 | 0,0194 | 7 | 0 |
| 235352_at | AI270356 | --- | 2,665245 | 0,0145 | 6 | 0 |
| 1552622_s_at | BQ613856 | POLR2J2 | 2,659574 | 0,0046 | 6 | 0 |
| 210942_s_at | AB022918 | ST3GAL6 | 2,621232 | 0,0169 | 6 | 0 |
| 219857_at | NM_024889 | C10orf81 | 2,615747 | 0,0152 | 6 | 0 |
| 218824_at | NM_018215 | FLJ10781 | 2,546473 | 0,0207 | 6 | 2 |
| 216733_s_at | X86401 | GATM | 2,51004 | 0,0211 | 6 | 0 |
| 219543_at | NM_022129 | MAWBP | 2,436054 | 0,0412 | 6 | 1 |
| 208092_s_at | NM_030797 | FAM49A | 2,416626 | 0,0248 | 6 | 1 |
| 226416_at | AL137679 | THEX1 | 2,413127 | 0,0415 | 7 | 0 |
| 203397_s_at | BF063271 | GALNT3 | 2,411382 | 0,0154 | 6 | 1 |
| 224027_at | AF110384 | CCL28 | 2,380952 | 0,0143 | 7 | 0 |
| 244872_at | BE514107 | SYNC1 | 2,359604 | 0,0429 | 6 | 1 |
| 226231_at | AI189509 | PAWR | 2,355713 | 0,0349 | 7 | 0 |
| 225338_at | AI767447 | ZYG11B | 2,352941 | 0,0226 | 6 | 0 |
| 1563498_s_at | AK090434 | LOC283130 | 2,334267 | 0,0356 | 6 | 0 |
| 213858_at | BE350026 | ZNF250 | 2,333178 | 0,0425 | 6 | 0 |
| 219397_at | NM_025147 | FLJ13448 | 2,311604 | 0,0479 | 6 | 1 |
| 243729_at | AI457984 | --- | 2,276867 | 0,0383 | 6 | 0 |
| 231984_at | BE958291 | MTAP | 2,2553 | 0,0706 | 6 | 2 |
| 214739_at | AI357539 | LRCH3 | 2,244669 | 0,0559 | 6 | 0 |
| 235142_at | AW006067 | ZBTB8 | 2,225189 | 0,0152 | 7 | 1 |
| 214129_at | AI821791 | PDE4DIP | 2,222222 | 0,0438 | 6 | 0 |
| 1558922_at | CA776505 | TIA1 | 2,212389 | 0,043 | 6 | 1 |
| 222163_s_at | BE890973 | SPATA5L1 | 2,164502 | 0,0346 | 7 | 2 |
| 215136_s_at | AL050353 | EXOSC8 | 2,138123 | 0,071 | 7 | 0 |
| 218685_s_at | NM_014311 | SMUG1 | 2,133561 | 0,0648 | 6 | 0 |
| 1557270_at | AA632049 | --- | 2,131742 | 0,0597 | 6 | 2 |
| 234295_at | AK000116 | DBR1 | 2,115059 | 0,0832 | 6 | 0 |
| 225288_at | AI949136 | COL27A1 | 2,110595 | 0,0437 | 7 | 0 |
| 202768_at | NM_006732 | FOSB | 2,103934 | 0,033 | 6 | 0 |
| 203790_s_at | N54448 | HRSP12 | 2,093364 | 0,0654 | 6 | 0 |
| 202250_s_at | NM_015726 | WDR42A | 2,091175 | 0,092 | 6 | 0 |
| 228111_s_at | AI004779 | DNAH1 | 2,086811 | 0,0597 | 6 | 1 |
| 232165_at | AL137725 | EPPK1 | 2,086376 | 0,0485 | 7 | 0 |
| 218486_at | AA149594 | KLF11 | 2,083767 | 0,0482 | 6 | 1 |
| 202672_s_at | NM_001674 | ATF3 | 2,081165 | 0,0443 | 6 | 0 |
| 215794_x_at | AC006144 | GLUD2 | 2,079434 | 0,0581 | 6 | 0 |
| 36711_at | AL021977 | MAFF | 2,076412 | 0,0267 | 7 | 1 |
| 1568807_a_at | AI301081 | NDFIP2 | 2,075981 | 0,023 | 6 | 1 |
| 226924_at | AI016355 | LOC400657 | 2,062706 | 0,0728 | 6 | 0 |
| 226426_at | BG149849 | ADNP | 2,057613 | 0,0564 | 6 | 0 |
| 213680_at | AI831452 | KRT6B | 2,04876 | 0,0217 | 8 | 0 |
| 229119_s_at | BG108034 | TTC19 | 2,04876 | 0,0703 | 6 | 1 |
| 230098_at | AW612407 | PHF20L1 | 2,024291 | 0,0629 | 6 | 2 |
| 213243_at | AI052003 | VPS13B | 2,023472 | 0,0626 | 6 | 1 |
| 242268_at | BE157991 | CUGBP2 | 2,020202 | 0,0281 | 6 | 2 |
| 222196_at | AK000470 | LOC286434 | 2,018978 | 0,0567 | 6 | 0 |
| 1552625_a_at | AB063105 | TRNT1 | 2,015723 | 0,0865 | 6 | 1 |
| 212338_at | AA621962 | MYO1D | 2,011263 | 0,0412 | 6 | 1 |
| 225040_s_at | AV699857 | RPE | 1,990842 | 0,0982 | 6 | 1 |
| 203625_x_at | BG105365 | SKP2 | 1,970443 | 0,0916 | 6 | 1 |
| 213056_at | AU145019 | FRMD4B | 1,969667 | 0,0229 | 6 | 1 |
| 227025_at | BG284497 | PPHLN1 | 1,949318 | 0,083 | 7 | 3 |
| 216248_s_at | S77154 | NR4A2 | 1,94439 | 0,0439 | 6 | 1 |
| 237839_at | BF433975 | ANK3 | 1,94439 | 0,0589 | 6 | 1 |
| 823_at | U84487 | CX3CL1 | 1,925298 | 0,0909 | 9 | 0 |
| 222450_at | AL035541 | TMEPAI | 1,905125 | 0,065 | 6 | 0 |
| 211686_s_at | AF251062 | RBM13 | 1,90295 | 0,0981 | 6 | 1 |
| 212277_at | AB014547 | MTMR4 | 1,893581 | 0,0887 | 6 | 1 |
| 226350_at | AU155565 | CHML | 1,889645 | 0,0614 | 6 | 1 |
| 218963_s_at | NM_015515 | KRT23 | 1,849112 | 0,0598 | 7 | 0 |
| 235451_at | AI439752 | SMAD5 | 1,78731 | 0,0805 | 7 | 2 |
| 205044_at | NM_014211 | GABRP | 1,77841 | 0,0435 | 6 | 1 |
| 49452_at | AI057637 | ACACB | 1,765537 | 0,0896 | 8 | 0 |
| 232752_at | AK001164 | LOXL1 | 1,377221 | 0,0959 | 6 | 1 |
| 211896_s_at | AF138302 | DCN | -2,0018 | 0,0947 | 1 | 7 |
| 207117_at | NM_015852 | H-plk | -2,4041 | 0,0885 | 1 | 6 |
| 202975_s_at | N21138 | RHOBTB3 | -2,4576 | 0,0963 | 1 | 7 |
| 203264_s_at | NM_015185 | ARHGEF9 | -2,5587 | 0,0907 | 0 | 6 |
| 209890_at | AF065389 | TSPAN5 | -2,5798 | 0,0344 | 0 | 7 |
| 212650_at | BF116032 | EHBP1 | -2,6373 | 0,0333 | 0 | 6 |
| 226760_at | BF666325 | LOC203411 | -3,0264 | 0,02 | 2 | 6 |

*FC, fold change for the specified comparison by rank products analysis; pfp, percentage of false positivities in rank products analysis; Increase – Decrease, count of changes more than 6 (out of 10) was used for pairwise comparisons of normal cells in between.*

Supplementary Table 2. Differentially expressed probe sets between ductal carcinoma (Tduc) and normal ductal (D) cells found by both rank products and pairwise analysis.

| Probe Set | Accession | Gene Symbol | FC:(Tduc/ normal ductal) | pfp | Increase | Decrease |
| --- | --- | --- | --- | --- | --- | --- |
| 209351_at | BC002690 | KRT14 | -30,9598 | 0 | 0 | 5 |
| 205157_s_at | NM_000422 | KRT17 | -21,3675 | 0 | 0 | 5 |
| 206378_at | NM_002411 | SCGB2A2 | -18,9753 | 0 | 1 | 4 |
| 204455_at | NM_001723 | DST | -14,8148 | 0 | 1 | 4 |
| 212236_x_at | Z19574 | KRT17 | -14,4092 | 0 | 0 | 5 |
| 204734_at | NM_002275 | KRT15 | -13,1926 | 0 | 0 | 5 |
| 209016_s_at | BC002700 | KRT7 | -12,8041 | 0 | 0 | 5 |
| 201820_at | NM_000424 | KRT5 | -12,7065 | 0 | 0 | 4 |
| 205363_at | NM_003986 | BBOX1 | -12,6582 | 0 | 0 | 4 |
| 211737_x_at | BC005916 | PTN | -12,5 | 0 | 0 | 5 |
| 229201_at | AW044658 | --- | -12,4844 | 0 | 0 | 5 |
| 206548_at | NM_024880 | FLJ23556 | -11,1732 | 0 | 0 | 5 |
| 224027_at | AF110384 | CCL28 | -11,0742 | 0 | 0 | 5 |
| 219857_at | NM_024889 | C10orf81 | -10,8932 | 0 | 1 | 4 |
| 1553602_at | NM_058173 | LOC118430 | -10,4167 | 0 | 0 | 4 |
| 212730_at | AK026420 | DMN | -10,395 | 0 | 0 | 5 |
| 214087_s_at | BF593509 | MYBPC1 | -9,99001 | 0 | 0 | 5 |
| 226147_s_at | AA838075 | PIGR | -9,95025 | 0 | 0 | 4 |
| 203296_s_at | NM_000702 | ATP1A2 | -9,56023 | 0 | 0 | 4 |
| 209395_at | M80927 | CHI3L1 | -9,49668 | 0 | 0 | 4 |
| 202037_s_at | NM_003012 | SFRP1 | -8,77193 | 0 | 0 | 4 |
| 205044_at | NM_014211 | GABRP | -8,67303 | 0 | 0 | 4 |
| 206157_at | NM_002852 | PTX3 | -8,52515 | 0 | 0 | 4 |
| 202036_s_at | AF017987 | SFRP1 | -8,39631 | 0 | 1 | 4 |
| 823_at | U84487 | CX3CL1 | -8,34028 | 0 | 0 | 5 |
| 209466_x_at | M57399 | PTN | -8,29187 | 0,0003 | 0 | 5 |
| 206799_at | NM_006551 | SCGB1D2 | -7,8064 | 0 | 1 | 4 |
| 214451_at | NM_003221 | TFAP2B | -7,73395 | 0 | 0 | 4 |
| 202672_s_at | NM_001674 | ATF3 | -7,68049 | 0 | 0 | 5 |
| 209278_s_at | L27624 | TFPI2 | -7,68049 | 0,0006 | 0 | 5 |
| 208004_at | NM_021225 | PROL1 | -7,59301 | 0,0006 | 0 | 5 |
| 229947_at | AI088609 | PI15 | -7,47943 | 0,0008 | 0 | 5 |
| 202935_s_at | AI382146 | SOX9 | -7,38552 | 0,0007 | 0 | 4 |
| 218963_s_at | NM_015515 | KRT23 | -7,37463 | 0,0009 | 0 | 5 |
| 204259_at | NM_002423 | MMP7 | -7,24638 | 0,0007 | 0 | 5 |
| 209291_at | AW157094 | ID4 | -7,08215 | 0,0006 | 0 | 4 |
| 221872_at | AI669229 | RARRES1 | -6,76133 | 0,0009 | 0 | 4 |
| 227702_at | AA557324 | CYP4X1 | -6,71592 | 0,0005 | 1 | 4 |
| 203951_at | NM_001299 | CNN1 | -6,58762 | 0,0013 | 0 | 5 |
| 235467_s_at | BF969982 | KCNC4 | -6,50618 | 0,0023 | 0 | 5 |
| 203400_s_at | NM_001063 | TF | -6,43501 | 0,0033 | 0 | 4 |
| 226755_at | AI375939 | --- | -6,32911 | 0,0021 | 0 | 4 |
| 222862_s_at | BG169832 | AK5 | -6,30517 | 0,0009 | 0 | 4 |
| 206032_at | AI797281 | DSC3 | -6,27353 | 0,0028 | 0 | 4 |
| 216005_at | BF434846 | TNC | -6,26959 | 0,0012 | 0 | 5 |
| 202768_at | NM_006732 | FOSB | -6,21504 | 0,0024 | 0 | 5 |
| 214651_s_at | U41813 | HOXA9 | -6,15764 | 0,0031 | 0 | 4 |
| 219768_at | NM_024626 | VTCN1 | -6,14251 | 0,0016 | 0 | 4 |
| 206392_s_at | NM_002888 | RARRES1 | -6,13874 | 0,0032 | 0 | 5 |
| 205051_s_at | NM_000222 | KIT | -6,13121 | 0,0031 | 0 | 4 |
| 227256_at | BG289456 | USP31 | -5,96659 | 0,003 | 0 | 4 |
| 225645_at | AI763378 | EHF | -5,93824 | 0,0032 | 0 | 4 |
| 1566539_at | AL050129 | LOC151877 | -5,85823 | 0,0033 | 0 | 4 |
| 235075_at | AI813438 | DSG3 | -5,8309 | 0,0043 | 0 | 4 |
| 205380_at | NM_002614 | PDZK1 | -5,82751 | 0,0039 | 0 | 5 |
| 214823_at | AF033199 | ZNF204 | -5,66572 | 0,0032 | 0 | 4 |
| 201496_x_at | S67238 | MYH11 | -5,65611 | 0,0038 | 0 | 4 |
| 214063_s_at | AI073407 | TF | -5,62114 | 0,0052 | 0 | 5 |
| 223623_at | AF325503 | ECRG4 | -5,60224 | 0,0028 | 0 | 4 |
| 206825_at | NM_000916 | OXTR | -5,53097 | 0,0005 | 1 | 4 |
| 230791_at | AU146924 | --- | -5,49149 | 0,0037 | 1 | 4 |
| 239258_at | BE551407 | RHOQ | -5,42594 | 0,0027 | 0 | 4 |
| 36711_at | AL021977 | MAFF | -5,41419 | 0,0048 | 0 | 5 |
| 213032_at | AI186739 | NFIB | -5,35906 | 0,0048 | 0 | 4 |
| 235521_at | AW137982 | HOXA3 | -5,30786 | 0,0062 | 0 | 4 |
| 214456_x_at | M23699 | SAA1 | -5,30504 | 0,0042 | 0 | 4 |
| 209170_s_at | AF016004 | GPM6B | -5,24109 | 0,0038 | 0 | 5 |
| 208370_s_at | NM_004414 | DSCR1 | -5,16262 | 0,0044 | 0 | 4 |
| 241421_at | N92599 | --- | -5,1573 | 0,0046 | 0 | 5 |
| 202274_at | NM_001615 | ACTG2 | -5,15198 | 0,0048 | 0 | 5 |
| 209292_at | AL022726 | ID4 | -5,14933 | 0,0025 | 0 | 4 |
| 202018_s_at | NM_002343 | LTF | -5,14403 | 0,0032 | 0 | 4 |
| 228748_at | AI653117 | CD59 | -5,14403 | 0,0061 | 0 | 4 |
| 213680_at | AI831452 | KRT6B | -5,08906 | 0,0044 | 0 | 4 |
| 227444_at | AW519141 | ARMCX4 | -4,99251 | 0,0044 | 0 | 5 |
| 213816_s_at | AA005141 | MET | -4,96771 | 0,0061 | 0 | 4 |
| 220625_s_at | AF115403 | ELF5 | -4,80077 | 0,006 | 1 | 4 |
| 228854_at | AI492388 | --- | -4,79157 | 0,0044 | 1 | 4 |
| 225540_at | BF342661 | MAP2 | -4,74834 | 0,006 | 0 | 4 |
| 203324_s_at | NM_001233 | CAV2 | -4,72144 | 0,0062 | 0 | 5 |
| 209270_at | L25541 | LAMB3 | -4,7081 | 0,006 | 1 | 4 |
| 213900_at | AA524029 | C9orf61 | -4,7081 | 0,0078 | 0 | 5 |
| 206552_s_at | NM_003182 | TAC1 | -4,69704 | 0,0062 | 0 | 4 |
| 203637_s_at | NM_000381 | MID1 | -4,68384 | 0,0057 | 0 | 4 |
| 202291_s_at | NM_000900 | MGP | -4,66853 | 0,0058 | 0 | 5 |
| 232882_at | AA079839 | FOXO1A | -4,64253 | 0,006 | 1 | 4 |
| 231149_s_at | AI828638 | ULK4 | -4,63607 | 0,0123 | 0 | 4 |
| 202035_s_at | AI332407 | SFRP1 | -4,60617 | 0,0121 | 0 | 4 |
| 224240_s_at | AF266504 | CCL28 | -4,59559 | 0,0119 | 0 | 5 |
| 214023_x_at | AL533838 | TUBB-PARALOG | -4,55581 | 0,0055 | 1 | 4 |
| 202504_at | NM_012101 | TRIM29 | -4,54339 | 0,0088 | 0 | 4 |
| 214598_at | AL049977 | CLDN8 | -4,54339 | 0,0206 | 0 | 4 |
| 237981_at | AA195941 | CMYA5 | -4,54133 | 0,0122 | 0 | 4 |
| 1558828_s_at | AL703532 | LOC401212 | -4,53721 | 0,0076 | 0 | 5 |
| 228143_at | AI684991 | --- | -4,53721 | 0,0101 | 0 | 4 |
| 223916_s_at | AF317392 | BCOR | -4,49438 | 0,013 | 0 | 4 |
| 239348_at | AI285970 | USP31 | -4,43262 | 0,0136 | 0 | 4 |
| 228885_at | AI862120 | MAMDC2 | -4,41306 | 0,0055 | 0 | 4 |
| 233520_s_at | AL359338 | CMYA5 | -4,36681 | 0,019 | 0 | 4 |
| 230815_at | AI684760 | LOC389765 | -4,3573 | 0,0141 | 0 | 5 |
| 236429_at | AI831874 | ZNF83 | -4,3535 | 0,0075 | 0 | 5 |
| 238481_at | AW512787 | MGP | -4,3535 | 0,0114 | 0 | 4 |
| 209189_at | BC004490 | FOS | -4,33276 | 0,0109 | 0 | 4 |
| 205016_at | NM_003236 | TGFA | -4,31406 | 0,0175 | 0 | 4 |
| 221556_at | BF792631 | CDC14B | -4,30663 | 0,0088 | 0 | 4 |
| 219492_at | NM_012110 | CHIC2 | -4,29738 | 0,0199 | 0 | 5 |
| 1556007_s_at | AI377389 | --- | -4,28633 | 0,0145 | 0 | 4 |
| 222529_at | BG251467 | SLC25A37 | -4,28633 | 0,0175 | 0 | 4 |
| 204846_at | NM_000096 | CP | -4,25713 | 0,0201 | 0 | 4 |
| 212724_at | BG054844 | RND3 | -4,24088 | 0,0182 | 0 | 4 |
| 221530_s_at | BE857425 | BHLHB3 | -4,2337 | 0,006 | 1 | 4 |
| 208127_s_at | NM_014011 | SOCS5 | -4,21941 | 0,0189 | 0 | 4 |
| 205816_at | NM_002214 | ITGB8 | -4,18235 | 0,0164 | 0 | 4 |
| 204591_at | NM_006614 | CHL1 | -4,16146 | 0,0162 | 1 | 4 |
| 209648_x_at | AL136896 | SOCS5 | -4,16146 | 0,0223 | 0 | 4 |
| 242208_at | AI634543 | --- | -4,15628 | 0,0247 | 0 | 5 |
| 201510_at | AF017307 | ELF3 | -4,09668 | 0,0202 | 0 | 4 |
| 243140_at | AI917901 | ACTA2 | -4,07498 | 0,0145 | 0 | 4 |
| 239381_at | AU155415 | KLK7 | -4,0568 | 0,0277 | 0 | 4 |
| 204636_at | NM_000494 | COL17A1 | -4,01123 | 0,0253 | 0 | 5 |
| 239301_at | BE551451 | RASA1 | -3,98724 | 0,0207 | 0 | 4 |
| 210942_s_at | AB022918 | ST3GAL6 | -3,97141 | 0,0246 | 0 | 4 |
| 208607_s_at | NM_030754 | SAA1 /// SAA2 | -3,94945 | 0,0191 | 0 | 5 |
| 213029_at | BG478428 | NFIB | -3,94633 | 0,02 | 0 | 5 |
| 209905_at | AI246769 | HOXA9 | -3,94322 | 0,0258 | 0 | 4 |
| 243016_at | AW271958 | ENOSF1 | -3,93236 | 0,0251 | 0 | 4 |
| 203687_at | NM_002996 | CX3CL1 | -3,90016 | 0,0272 | 0 | 5 |
| 226301_at | AV729072 | C6orf192 | -3,85951 | 0,0106 | 1 | 4 |
| 1556006_s_at | BQ025347 | --- | -3,84172 | 0,0082 | 1 | 4 |
| 204537_s_at | NM_004961 | GABRE | -3,84025 | 0,0244 | 0 | 4 |
| 1555847_a_at | BU617052 | LOC284454 | -3,83142 | 0,0329 | 0 | 4 |
| 202342_s_at | NM_015271 | TRIM2 | -3,82555 | 0,0173 | 0 | 5 |
| 235497_at | AL079648 | LOC284591 | -3,82409 | 0,0256 | 0 | 4 |
| 213355_at | AI989567 | ST3GAL6 | -3,79651 | 0,0415 | 0 | 4 |
| 209621_s_at | AF002280 | PDLIM3 | -3,79363 | 0,0268 | 0 | 4 |
| 220230_s_at | NM_016229 | CYB5R2 | -3,77074 | 0,0384 | 0 | 5 |
| 227662_at | AA541622 | SYNPO2 | -3,76506 | 0,0165 | 1 | 4 |
| 228686_at | BE217923 | --- | -3,76364 | 0,0278 | 0 | 4 |
| 238584_at | W52934 | IQCA | -3,75516 | 0,0199 | 1 | 4 |
| 202267_at | NM_005562 | LAMC2 | -3,73972 | 0,03 | 0 | 5 |
| 201161_s_at | NM_003651 | CSDA | -3,72995 | 0,0255 | 0 | 4 |
| 225728_at | AI659533 | --- | -3,7092 | 0,0377 | 0 | 5 |
| 204041_at | NM_000898 | MAOB | -3,69822 | 0,0231 | 0 | 4 |
| 202888_s_at | NM_001150 | ANPEP | -3,69276 | 0,0149 | 1 | 4 |
| 214967_at | AU146983 | --- | -3,68053 | 0,0251 | 1 | 4 |
| 201160_s_at | AL556190 | CSDA | -3,67918 | 0,0256 | 0 | 4 |
| 229170_s_at | AW024437 | TTC18 | -3,65364 | 0,0297 | 0 | 4 |
| 204607_at | NM_005518 | HMGCS2 | -3,62845 | 0,0155 | 0 | 5 |
| 227183_at | AI417267 | LOC401212 | -3,62713 | 0,0385 | 0 | 4 |
| 201693_s_at | AV733950 | EGR1 | -3,60101 | 0,0261 | 0 | 5 |
| 218706_s_at | AW575493 | NS3TP2 | -3,57398 | 0,0286 | 0 | 4 |
| 230661_at | AW451999 | --- | -3,52237 | 0,0328 | 0 | 4 |
| 206453_s_at | NM_016250 | NDRG2 | -3,51865 | 0,04 | 0 | 5 |
| 241991_at | AI629041 | RP3-473B4.1 | -3,50877 | 0,0379 | 0 | 4 |
| 226171_at | BF111925 | FLJ20209 | -3,50631 | 0,0385 | 0 | 4 |
| 244107_at | AW189097 | --- | -3,50385 | 0,0558 | 0 | 4 |
| 215992_s_at | AL117397 | RAPGEF2 | -3,49406 | 0,0451 | 0 | 4 |
| 236752_at | AA913146 | PKP4 | -3,4904 | 0,0477 | 0 | 4 |
| 228482_at | AV702789 | NBLA10383 | -3,48189 | 0,0375 | 0 | 4 |
| 201012_at | NM_000700 | ANXA1 | -3,47705 | 0,0324 | 0 | 4 |
| 204803_s_at | NM_004165 | RRAD | -3,47584 | 0,0443 | 0 | 4 |
| 1563498_s_at | AK090434 | LOC283130 | -3,47102 | 0,0384 | 0 | 5 |
| 229357_at | BF060767 | ADAMTS5 | -3,46861 | 0,0441 | 0 | 4 |
| 203706_s_at | NM_003507 | FZD7 | -3,44947 | 0,0402 | 1 | 4 |
| 226179_at | N63920 | SLC25A37 | -3,44471 | 0,0474 | 0 | 5 |
| 226380_at | N21442 | PTPN21 | -3,43879 | 0,0474 | 0 | 4 |
| 226863_at | AI674565 | --- | -3,43761 | 0,0534 | 0 | 4 |
| 1555898_at | BQ003366 | LOC150759 | -3,43171 | 0,0584 | 0 | 4 |
| 229633_at | AA115512 | C8orf35 | -3,43053 | 0,0399 | 0 | 4 |
| 203256_at | NM_001793 | CDH3 | -3,42818 | 0,039 | 0 | 4 |
| 201681_s_at | AB011155 | DLG5 | -3,427 | 0,0536 | 0 | 5 |
| 215033_at | AI189753 | TM4SF1 | -3,42583 | 0,0422 | 1 | 4 |
| 1565162_s_at | D16947 | MGST1 | -3,42114 | 0,0328 | 1 | 4 |
| 224823_at | AA526844 | MYLK | -3,41647 | 0,0363 | 1 | 4 |
| 229842_at | AA527180 | ELF3 | -3,4118 | 0,0563 | 0 | 5 |
| 238049_at | AW971198 | NS3TP2 | -3,4118 | 0,0468 | 0 | 4 |
| 212592_at | AV733266 | IGJ | -3,40716 | 0,016 | 1 | 4 |
| 202083_s_at | AI017770 | SEC14L1 | -3,40136 | 0,0379 | 0 | 4 |
| 203180_at | NM_000693 | ALDH1A3 | -3,37268 | 0,044 | 0 | 4 |
| 1556666_a_at | BU680030 | TTC6 | -3,35008 | 0,0378 | 1 | 4 |
| 206391_at | NM_002888 | RARRES1 | -3,32779 | 0,0602 | 0 | 4 |
| 236635_at | AI332774 | ZNF667 | -3,32668 | 0,0548 | 0 | 5 |
| 207961_x_at | NM_022870 | MYH11 | -3,32447 | 0,0442 | 0 | 4 |
| 232584_at | AU147926 | --- | -3,30688 | 0,0428 | 1 | 4 |
| 1557051_s_at | CA448125 | --- | -3,29598 | 0,0448 | 0 | 4 |
| 239130_at | AA905821 | --- | -3,28839 | 0,0594 | 0 | 4 |
| 213526_s_at | BF215644 | F25965 | -3,27225 | 0,0451 | 0 | 4 |
| 230493_at | AW664964 | TMEM46 | -3,27225 | 0,0145 | 1 | 4 |
| 226848_at | AI571166 | NR2C2 | -3,25627 | 0,055 | 0 | 5 |
| 228335_at | AW264204 | CLDN11 | -3,25203 | 0,047 | 0 | 4 |
| 202833_s_at | NM_000295 | SERPINA1 | -3,23625 | 0,0557 | 1 | 4 |
| 1553111_a_at | NM_152903 | KBTBD6 | -3,23415 | 0,0632 | 0 | 4 |
| 209283_at | AF007162 | CRYAB | -3,22997 | 0,0527 | 0 | 4 |
| 218736_s_at | NM_017734 | PALMD | -3,22581 | 0,0565 | 0 | 5 |
| 203636_at | BE967532 | MID1 | -3,22373 | 0,0342 | 1 | 4 |
| 239250_at | BE966038 | ZNF542 | -3,20616 | 0,0603 | 0 | 4 |
| 209289_at | AI700518 | NFIB | -3,2041 | 0,0571 | 0 | 4 |
| 226575_at | T89120 | ZNF462 | -3,20102 | 0,0646 | 0 | 4 |
| 203178_at | NM_001482 | GATM | -3,19591 | 0,0515 | 1 | 4 |
| 221194_s_at | NM_016125 | LOC51136 | -3,19387 | 0,0736 | 0 | 4 |
| 218424_s_at | NM_018234 | STEAP3 | -3,17662 | 0,0746 | 0 | 4 |
| 1558922_at | CA776505 | TIA1 | -3,16256 | 0,0566 | 0 | 4 |
| 208399_s_at | NM_000114 | EDN3 | -3,15657 | 0,0256 | 0 | 4 |
| 233558_s_at | AK023390 | FLJ12716 | -3,14861 | 0,0518 | 0 | 4 |
| 228156_at | AW342078 | --- | -3,13972 | 0,062 | 0 | 4 |
| 232360_at | AA565141 | EHF | -3,13578 | 0,0625 | 0 | 4 |
| 225344_at | AL035689 | NCOA7 | -3,1348 | 0,0663 | 0 | 4 |
| 1568780_at | BC030211 | LOC497257 | -3,12793 | 0,0593 | 0 | 4 |
| 235099_at | AW080832 | CKLFSF8 | -3,125 | 0,0784 | 1 | 4 |
| 209220_at | L47125 | GPC3 | -3,12207 | 0,0629 | 0 | 4 |
| 227272_at | BE673226 | FLJ43339 | -3,11721 | 0,0884 | 0 | 5 |
| 202555_s_at | NM_005965 | MYLK | -3,11526 | 0,0619 | 0 | 4 |
| 232034_at | AL117607 | LOC203274 | -3,11526 | 0,0598 | 0 | 4 |
| 204288_s_at | NM_021069 | SORBS2 | -3,11236 | 0,0727 | 0 | 4 |
| 244521_at | BG236742 | ZNF218 | -3,10463 | 0,0621 | 0 | 4 |
| 203854_at | NM_000204 | IF | -3,09981 | 0,0669 | 0 | 4 |
| 210172_at | D26121 | SF1 | -3,09023 | 0,0597 | 0 | 5 |
| 204990_s_at | NM_000213 | ITGB4 | -3,08833 | 0,0585 | 0 | 4 |
| 207836_s_at | NM_006867 | RBPMS | -3,06654 | 0,0595 | 0 | 4 |
| 227148_at | AI913749 | PLEKHH2 | -3,0553 | 0,0692 | 0 | 4 |
| 240458_at | AI242023 | ITPR2 | -3,05344 | 0,0613 | 0 | 5 |
| 215223_s_at | W46388 | SOD2 | -3,04136 | 0,0786 | 0 | 4 |
| 209295_at | AF016266 | TNFRSF10B | -3,03674 | 0,0668 | 0 | 4 |
| 222528_s_at | BG251467 | SLC25A37 | -3,03674 | 0,0778 | 0 | 4 |
| 209800_at | AF061812 | KRT16 | -3,02297 | 0,0884 | 0 | 4 |
| 203726_s_at | NM_000227 | LAMA3 | -3,02206 | 0,0779 | 0 | 5 |
| 201694_s_at | NM_001964 | EGR1 | -3,02024 | 0,0664 | 0 | 4 |
| 217022_s_at | S55735 | IGHA1 /// IGHA2 /// MGC27165 | -3,00391 | 0,0455 | 1 | 4 |
| 243030_at | AA211369 | MAP3K1 | -3,00391 | 0,069 | 0 | 4 |
| 205382_s_at | NM_001928 | DF | -2,97619 | 0,0669 | 0 | 4 |
| 212715_s_at | AB020626 | MICAL3 | -2,95683 | 0,0746 | 0 | 4 |
| 210074_at | AF070448 | CTSL2 | -2,95247 | 0,0825 | 0 | 4 |
| 226837_at | BE967019 | SPRED1 | -2,93169 | 0,0966 | 0 | 4 |
| 205547_s_at | NM_003186 | TAGLN | -2,92997 | 0,0788 | 0 | 4 |
| 226435_at | AU145309 | PAPLN | -2,91971 | 0,0522 | 1 | 4 |
| 226607_at | AI498144 | C20orf194 | -2,9163 | 0,0894 | 0 | 4 |
| 208498_s_at | NM_004038 | AMY1A /// AMY1B /// AMY1C /// AMY2A /// AMY2B | -2,9036 | 0,0783 | 0 | 4 |
| 225724_at | AW136120 | FLJ31306 | -2,89855 | 0,0703 | 1 | 4 |
| 211458_s_at | AF180519 | GABARAPL1 /// GABARAPL3 | -2,88767 | 0,0901 | 0 | 5 |
| 231987_at | AW081196 | LOC401212 | -2,88767 | 0,0938 | 0 | 4 |
| 218541_s_at | NM_020130 | C8orf4 | -2,87522 | 0,045 | 1 | 4 |
| 201525_at | NM_001647 | APOD | -2,87109 | 0,0598 | 0 | 4 |
| 213238_at | AI478147 | ATP10D | -2,86862 | 0,0468 | 1 | 4 |
| 212463_at | BE379006 | CD59 | -2,86697 | 0,0971 | 0 | 5 |
| 237180_at | T97717 | PSME4 | -2,85714 | 0,0823 | 0 | 4 |
| 232762_at | AU146385 | KIAA1217 | -2,85144 | 0,0938 | 0 | 4 |
| 209129_at | AF000974 | TRIP6 | -2,8401 | 0,0786 | 0 | 4 |
| 244297_at | AI806762 | FLJ35740 | -2,83849 | 0,0785 | 0 | 4 |
| 1555882_at | AJ271379 | SPIN3 | -2,8177 | 0,0635 | 1 | 4 |
| 226869_at | AI655611 | EGFL3 | -2,8177 | 0,0814 | 0 | 5 |
| 228653_at | AI700341 | LOC389432 | -2,8177 | 0,0785 | 0 | 4 |
| 219630_at | NM_005764 | PDZK1IP1 | -2,81373 | 0,0965 | 0 | 4 |
| 244845_at | BF725383 | FOXP1 | -2,80112 | 0,0787 | 1 | 4 |
| 202861_at | NM_002616 | PER1 | -2,79564 | 0,0787 | 0 | 4 |
| 201983_s_at | AW157070 | EGFR | -2,79018 | 0,0679 | 1 | 4 |
| 235419_at | AW612461 | ERRFI1 | -2,76243 | 0,0697 | 1 | 4 |
| 241763_s_at | BF244402 | --- | -2,74952 | 0,0679 | 1 | 4 |
| 201041_s_at | NM_004417 | DUSP1 | -2,74348 | 0,0892 | 0 | 5 |
| 1555925_at | BE045368 | --- | -2,73598 | 0,0966 | 0 | 4 |
| 209183_s_at | AL136653 | C10orf10 | -2,71003 | 0,0871 | 0 | 5 |
| 218980_at | NM_025135 | FHOD3 | -2,68528 | 0,0863 | 1 | 4 |
| 235766_x_at | AA743462 | EIF2C2 | -2,64061 | 0,0903 | 1 | 4 |
| 236359_at | AW026241 | SCN4B | -2,61233 | 0,0982 | 1 | 4 |
| 243241_at | AW341473 | LOC400880 /// LOC440155 /// LOC440161 | -2,0555 | 0,0381 | 1 | 4 |
| 1553622_a_at | NM_152597 | FSIP1 | 2,8516 | 0,0802 | 4 | 0 |
| 229800_at | AI129626 | DCAMKL1 | 2,9059 | 0,0557 | 4 | 1 |
| 219015_s_at | NM_018466 | GLT28D1 | 2,9395 | 0,0996 | 4 | 0 |
| 238822_at | AI743753 | MRPL3 | 2,9818 | 0,072 | 4 | 0 |
| 223243_s_at | BF439488 | C1orf22 | 2,9855 | 0,0982 | 4 | 0 |
| 230165_at | N31731 | SGOL2 | 3,009 | 0,0826 | 4 | 0 |
| 214764_at | AW029169 | KIAA0507 | 3,0765 | 0,0989 | 4 | 1 |
| 206648_at | NM_016536 | ZNF571 | 3,1047 | 0,0809 | 4 | 0 |
| 226899_at | AK022859 | UNC5B | 3,1314 | 0,076 | 4 | 0 |
| 204320_at | NM_001854 | COL11A1 | 3,162 | 0,0492 | 4 | 0 |
| 211980_at | AI922605 | COL4A1 | 3,1886 | 0,0686 | 4 | 0 |
| 203008_x_at | NM_005783 | TXNDC9 | 3,258 | 0,0957 | 4 | 0 |
| 204170_s_at | NM_001827 | CKS2 | 3,2825 | 0,0551 | 4 | 1 |
| 211758_x_at | BC005968 | TXNDC9 | 3,2924 | 0,0912 | 4 | 0 |
| 236922_at | AA772352 | --- | 3,319 | 0,0968 | 4 | 1 |
| 211161_s_at | AF130082 | COL3A1 | 3,3245 | 0,0906 | 4 | 0 |
| 207828_s_at | NM_005196 | CENPF | 3,4218 | 0,0764 | 4 | 0 |
| 230893_at | AI223870 | DNAJA5 | 3,5131 | 0,0486 | 4 | 0 |
| 223122_s_at | AF311912 | SFRP2 | 3,5534 | 0,073 | 4 | 0 |
| 229479_at | AI739132 | --- | 3,5763 | 0,02 | 4 | 0 |
| 240145_at | AW628059 | --- | 3,589 | 0,0405 | 4 | 0 |
| 228316_at | AA905470 | FLJ31438 | 3,7456 | 0,0198 | 4 | 0 |
| 212354_at | BE500977 | SULF1 | 3,7993 | 0,0319 | 5 | 0 |
| 211421_s_at | M31213 | RET | 3,8028 | 0,006 | 4 | 0 |
| 201695_s_at | NM_000270 | NP | 3,9177 | 0,0288 | 4 | 0 |
| 211764_s_at | BC005980 | UBE2D1 | 3,9307 | 0,0285 | 4 | 0 |
| 235476_at | AW182459 | TRIM59 | 3,9432 | 0,0287 | 5 | 0 |
| 220146_at | NM_016562 | TLR7 | 3,9976 | 0,0104 | 4 | 0 |
| 212353_at | AI479175 | SULF1 | 4,0321 | 0,0082 | 4 | 0 |
| 1556499_s_at | BE221212 | COL1A1 | 4,0681 | 0,0267 | 5 | 0 |
| 217735_s_at | AW007368 | EIF2AK1 | 4,0825 | 0,015 | 4 | 0 |
| 225267_at | AI935246 | KPNA4 | 4,1092 | 0,0256 | 5 | 0 |
| 201744_s_at | NM_002345 | LUM | 4,1172 | 0,0409 | 4 | 0 |
| 212344_at | AW043713 | SULF1 | 4,2708 | 0,0149 | 5 | 0 |
| 239108_at | H16791 | MLSTD1 | 4,3579 | 0,0126 | 4 | 0 |
| 221729_at | AL575735 | COL5A2 | 4,3715 | 0,0097 | 4 | 0 |
| 211776_s_at | BC006141 | EPB41L3 | 4,3877 | 0,0116 | 4 | 0 |
| 226760_at | BF666325 | LOC203411 | 4,4856 | 0,0115 | 5 | 0 |
| 219087_at | NM_017680 | ASPN | 4,5494 | 0,019 | 4 | 0 |
| 218986_s_at | NM_017631 | FLJ20035 | 4,5542 | 0,0212 | 4 | 0 |
| 218585_s_at | NM_016448 | DTL | 4,82 | 0,005 | 4 | 0 |
| 204620_s_at | NM_004385 | CSPG2 | 4,8986 | 0,01 | 4 | 0 |
| 210495_x_at | AF130095 | FN1 | 4,9833 | 0,0104 | 4 | 0 |
| 212464_s_at | X02761 | FN1 | 5,0085 | 0,0108 | 4 | 0 |
| 202403_s_at | AA788711 | COL1A2 | 5,0141 | 0,0104 | 4 | 0 |
| 202766_s_at | NM_000138 | FBN1 | 5,0184 | 0,0104 | 4 | 0 |
| 210809_s_at | D13665 | POSTN | 5,06 | 0,0095 | 4 | 0 |
| 202310_s_at | K01228 | COL1A1 | 5,1365 | 0,0057 | 5 | 0 |
| 216442_x_at | AK026737 | FN1 | 5,2139 | 0,01 | 4 | 0 |
| 204619_s_at | BF590263 | CSPG2 | 5,2272 | 0,0083 | 4 | 0 |
| 221731_x_at | BF218922 | CSPG2 | 5,3351 | 0,0089 | 4 | 0 |
| 217523_at | AV700298 | CD44 | 5,3921 | 0,0088 | 4 | 0 |
| 201291_s_at | AU159942 | TOP2A | 5,5987 | 0,0022 | 4 | 0 |
| 205941_s_at | AI376003 | COL10A1 | 5,6492 | 0,0029 | 4 | 0 |
| 211719_x_at | BC005858 | FN1 | 5,6992 | 0,0054 | 4 | 0 |
| 213909_at | AU147799 | LRRC15 | 5,7103 | 0,0017 | 4 | 0 |
| 238681_at | R46180 | GDPD1 | 5,8292 | 0,0025 | 4 | 1 |
| 202311_s_at | AI743621 | COL1A1 | 6,0447 | 0,002 | 4 | 0 |
| 215646_s_at | R94644 | CSPG2 | 6,7945 | 0 | 5 | 0 |
| 37892_at | J04177 | COL11A1 | 7,783 | 0 | 5 | 0 |
| 1555778_a_at | AY140646 | POSTN | 9,8216 | 0 | 4 | 0 |
| 202404_s_at | NM_000089 | COL1A2 | 10,053 | 0 | 5 | 0 |

*FC, fold change for the specified comparison by rank products analysis; pfp, percentage of false positivities in rank products analysis; Increase – Decrease, count of changes more than 4 (out of 5) was used for parwise comparisons between tumour and normal cells.*

Supplementary Table 3. Differentially expressed probe sets between ductal carcinoma (Tduc) and normal lobular (L) cells found by both rank products and pairwise analysis.

| Probe Set | Accession | Gene Symbol | FC:(Tduc/ normal lobular) | pfp | Increase | Decrease |
| --- | --- | --- | --- | --- | --- | --- |
| 209351_at | BC002690 | KRT14 | -22,8833 | 0 | 0 | 4 |
| 205157_s_at | NM_000422 | KRT17 | -17,6991 | 0 | 0 | 4 |
| 206378_at | NM_002411 | SCGB2A2 | -12,1507 | 0 | 1 | 4 |
| 212730_at | AK026420 | DMN | -9,96016 | 0 | 0 | 5 |
| 201820_at | NM_000424 | KRT5 | -8,86525 | 0 | 1 | 4 |
| 203296_s_at | NM_000702 | ATP1A2 | -8,23723 | 0 | 0 | 4 |
| 202036_s_at | AF017987 | SFRP1 | -7,47943 | 0 | 0 | 4 |
| 202037_s_at | NM_003012 | SFRP1 | -7,12251 | 0 | 0 | 5 |
| 211737_x_at | BC005916 | PTN | -7,06714 | 0 | 0 | 4 |
| 1553602_at | NM_058173 | LOC118430 | -7,05716 | 0 | 0 | 4 |
| 203951_at | NM_001299 | CNN1 | -6,71592 | 0 | 0 | 5 |
| 223623_at | AF325503 | ECRG4 | -6,57895 | 0,0006 | 0 | 4 |
| 229947_at | AI088609 | PI15 | -6,50195 | 0,0013 | 0 | 4 |
| 206548_at | NM_024880 | FLJ23556 | -6,34115 | 0,001 | 0 | 5 |
| 229201_at | AW044658 | --- | -5,84454 | 0,0044 | 0 | 5 |
| 209466_x_at | M57399 | PTN | -5,77701 | 0,0015 | 0 | 5 |
| 209291_at | AW157094 | ID4 | -5,13875 | 0,0027 | 0 | 5 |
| 204855_at | NM_002639 | SERPINB5 | -5,13347 | 0,0054 | 0 | 4 |
| 204304_s_at | NM_006017 | PROM1 | -4,97512 | 0,0016 | 1 | 4 |
| 223475_at | AF142573 | CRISPLD1 | -4,97018 | 0,0054 | 0 | 4 |
| 226755_at | AI375939 | --- | -4,94805 | 0,0102 | 0 | 4 |
| 1558828_s_at | AL703532 | LOC401212 | -4,93827 | 0,006 | 0 | 4 |
| 201496_x_at | S67238 | MYH11 | -4,89476 | 0,0047 | 1 | 4 |
| 226147_s_at | AA838075 | PIGR | -4,80307 | 0,0129 | 0 | 4 |
| 209170_s_at | AF016004 | GPM6B | -4,7824 | 0,0055 | 0 | 4 |
| 202291_s_at | NM_000900 | MGP | -4,60193 | 0,0064 | 0 | 4 |
| 214456_x_at | M23699 | SAA1 | -4,54752 | 0,0046 | 0 | 4 |
| 204259_at | NM_002423 | MMP7 | -4,51671 | 0,0186 | 0 | 4 |
| 202672_s_at | NM_001674 | ATF3 | -4,48833 | 0,0047 | 0 | 5 |
| 205380_at | NM_002614 | PDZK1 | -4,46229 | 0,0143 | 0 | 4 |
| 1556006_s_at | BQ025347 | --- | -4,29 | 0,0087 | 0 | 4 |
| 202274_at | NM_001615 | ACTG2 | -4,23729 | 0,0122 | 0 | 4 |
| 204389_at | NM_000240 | MAOA | -4,18936 | 0,0145 | 0 | 4 |
| 205044_at | NM_014211 | GABRP | -4,15455 | 0,0137 | 1 | 4 |
| 243140_at | AI917901 | ACTA2 | -4,02414 | 0,0184 | 0 | 4 |
| 205382_s_at | NM_001928 | DF | -3,96511 | 0,0183 | 0 | 5 |
| 220625_s_at | AF115403 | ELF5 | -3,9604 | 0,015 | 0 | 4 |
| 209613_s_at | M21692 | ADH1B | -3,93391 | 0,0052 | 1 | 4 |
| 223600_s_at | AL136867 | KIAA1683 | -3,69822 | 0,0281 | 0 | 4 |
| 228885_at | AI862120 | MAMDC2 | -3,65764 | 0,0279 | 0 | 4 |
| 222862_s_at | BG169832 | AK5 | -3,64299 | 0,0233 | 0 | 4 |
| 823_at | U84487 | CX3CL1 | -3,62188 | 0,043 | 0 | 4 |
| 202768_at | NM_006732 | FOSB | -3,61141 | 0,0353 | 0 | 5 |
| 218087_s_at | NM_015385 | SORBS1 | -3,59971 | 0,0508 | 0 | 4 |
| 220230_s_at | NM_016229 | CYB5R2 | -3,56633 | 0,0466 | 0 | 5 |
| 221796_at | AA707199 | NTRK2 | -3,51741 | 0,0182 | 1 | 4 |
| 213029_at | BG478428 | NFIB | -3,51741 | 0,0274 | 0 | 4 |
| 224823_at | AA526844 | MYLK | -3,51617 | 0,038 | 0 | 4 |
| 201510_at | AF017307 | ELF3 | -3,48311 | 0,0491 | 0 | 4 |
| 209031_at | AL519710 | IGSF4 | -3,38639 | 0,0678 | 0 | 4 |
| 209189_at | BC004490 | FOS | -3,3761 | 0,0375 | 0 | 4 |
| 205498_at | NM_000163 | GHR | -3,36361 | 0,0794 | 0 | 5 |
| 225724_at | AW136120 | FLJ31306 | -3,35909 | 0,0359 | 0 | 4 |
| 233884_at | AL512747 | HIVEP3 | -3,35458 | 0,0793 | 0 | 4 |
| 238481_at | AW512787 | MGP | -3,33333 | 0,0232 | 0 | 4 |
| 212741_at | AA923354 | MAOA | -3,30469 | 0,0382 | 0 | 4 |
| 205547_s_at | NM_003186 | TAGLN | -3,27654 | 0,0465 | 0 | 4 |
| 1554636_at | BC032569 | --- | -3,26371 | 0,0866 | 0 | 5 |
| 214823_at | AF033199 | ZNF204 | -3,24359 | 0,0537 | 0 | 4 |
| 205883_at | NM_006006 | ZBTB16 | -3,16156 | 0,0261 | 0 | 4 |
| 214790_at | AK001406 | SENP6 | -3,1211 | 0,0907 | 0 | 4 |
| 220425_x_at | NM_017578 | ROPN1B | -3,10655 | 0,0491 | 0 | 4 |
| 213238_at | AI478147 | ATP10D | -3,09885 | 0,0412 | 0 | 4 |
| 209283_at | AF007162 | CRYAB | -3,08261 | 0,0599 | 0 | 5 |
| 216614_at | AL049988 | ITPR2 | -3,06937 | 0,0674 | 0 | 4 |
| 241017_at | AA779927 | TBC1D8 | -3,06091 | 0,0824 | 0 | 4 |
| 201160_s_at | AL556190 | CSDA | -3,05904 | 0,0532 | 1 | 4 |
| 219857_at | NM_024889 | C10orf81 | -3,0553 | 0,0537 | 1 | 4 |
| 232541_at | AK000106 | EGFR | -3,03951 | 0,0966 | 0 | 4 |
| 207961_x_at | NM_022870 | MYH11 | -3,03674 | 0,0615 | 0 | 4 |
| 238584_at | W52934 | IQCA | -3,03398 | 0,0344 | 1 | 4 |
| 227239_at | AV734839 | DRCTNNB1A | -3,01296 | 0,0794 | 0 | 4 |
| 217627_at | BE515346 | ZNF573 | -3,003 | 0,0937 | 0 | 4 |
| 203065_s_at | NM_001753 | CAV1 | -2,93083 | 0,056 | 0 | 4 |
| 209292_at | AL022726 | ID4 | -2,9163 | 0,0383 | 0 | 5 |
| 217022_s_at | S55735 | IGHA1 /// IGHA2 /// MGC27165 | -2,886 | 0,0603 | 1 | 4 |
| 238625_at | AI452457 | C1orf168 | -2,86041 | 0,0819 | 1 | 4 |
| 201012_at | NM_000700 | ANXA1 | -2,84576 | 0,0737 | 0 | 5 |
| 227662_at | AA541622 | SYNPO2 | -2,83768 | 0,0674 | 1 | 4 |
| 221234_s_at | NM_021813 | BACH2 | -2,78629 | 0,0431 | 1 | 4 |
| 229623_at | BF508344 | LOC441027 | -2,74348 | 0,0998 | 1 | 4 |
| 239258_at | BE551407 | RHOQ | -2,68601 | 0,0948 | 0 | 4 |
| 225895_at | AI634580 | SYNPO2 | -2,6617 | 0,0962 | 1 | 4 |
| 208370_s_at | NM_004414 | DSCR1 | -2,65604 | 0,0899 | 0 | 5 |
| 218197_s_at | NM_018002 | OXR1 | -2,64901 | 0,0982 | 0 | 4 |
| 222113_s_at | AV710549 | EPS15L1 | 2,6948 | 0,0801 | 4 | 0 |
| 227245_at | AW511198 | FLJ13089 | 2,7297 | 0,0955 | 4 | 0 |
| 204825_at | NM_014791 | MELK | 2,7654 | 0,0813 | 4 | 0 |
| 222250_s_at | AK001363 | C1orf73 | 2,7871 | 0,1002 | 4 | 0 |
| 224377_s_at | AF274957 | RAB18 | 2,8636 | 0,0901 | 5 | 0 |
| 222608_s_at | AK023208 | ANLN | 2,8652 | 0,0763 | 4 | 0 |
| 220329_s_at | NM_017909 | C6orf96 | 2,8849 | 0,086 | 4 | 0 |
| 229479_at | AI739132 | --- | 2,8972 | 0,0633 | 4 | 0 |
| 208091_s_at | NM_030796 | ECOP | 2,9726 | 0,0915 | 4 | 0 |
| 212344_at | AW043713 | SULF1 | 2,9789 | 0,0816 | 5 | 0 |
| 206710_s_at | NM_012307 | EPB41L3 | 2,9915 | 0,0937 | 4 | 0 |
| 222555_s_at | AI338045 | MRPL44 | 3,0078 | 0,073 | 4 | 0 |
| 213007_at | W74442 | FLJ10719 | 3,0104 | 0,0803 | 4 | 0 |
| 223746_at | BC005231 | STK4 | 3,0133 | 0,0904 | 4 | 1 |
| 205034_at | NM_004702 | CCNE2 | 3,0135 | 0,0575 | 4 | 0 |
| 212989_at | AI377497 | TMEM23 | 3,0338 | 0,0767 | 4 | 0 |
| 222754_at | BE552215 | TRNT1 | 3,0363 | 0,0759 | 4 | 0 |
| 204620_s_at | NM_004385 | CSPG2 | 3,0416 | 0,0795 | 4 | 0 |
| 239108_at | H16791 | MLSTD1 | 3,0496 | 0,091 | 4 | 0 |
| 211421_s_at | M31213 | RET | 3,0784 | 0,0153 | 4 | 0 |
| 204051_s_at | AW089415 | SFRP4 | 3,1089 | 0,0831 | 4 | 1 |
| 212905_at | AI872408 | CSTF2T | 3,1102 | 0,0292 | 4 | 1 |
| 242049_s_at | BE783098 | NAG | 3,117 | 0,0805 | 4 | 0 |
| 220338_at | NM_018037 | RALGPS2 | 3,189 | 0,0492 | 4 | 0 |
| 238822_at | AI743753 | MRPL3 | 3,2004 | 0,0406 | 4 | 1 |
| 222471_s_at | AI743396 | KCMF1 | 3,2026 | 0,0487 | 4 | 0 |
| 226899_at | AK022859 | UNC5B | 3,217 | 0,0483 | 5 | 0 |
| 209080_x_at | AF118652 | TXNL2 | 3,2585 | 0,0514 | 5 | 0 |
| 224751_at | BE738276 | --- | 3,2615 | 0,0569 | 4 | 0 |
| 235653_s_at | BF685315 | THAP6 | 3,3428 | 0,0782 | 4 | 0 |
| 229218_at | AA628535 | COL1A2 | 3,3687 | 0,0487 | 5 | 0 |
| 203988_s_at | NM_004480 | FUT8 | 3,4031 | 0,0492 | 5 | 0 |
| 214764_at | AW029169 | KIAA0507 | 3,4072 | 0,0554 | 4 | 0 |
| 219673_at | NM_017696 | C6orf61 | 3,431 | 0,0468 | 4 | 1 |
| 229450_at | AI075407 | IFIT3 | 3,4488 | 0,048 | 4 | 0 |
| 228032_s_at | AW071458 | --- | 3,4537 | 0,0481 | 4 | 0 |
| 222077_s_at | AU153848 | RACGAP1 | 3,4643 | 0,0306 | 4 | 0 |
| 221731_x_at | BF218922 | CSPG2 | 3,4714 | 0,0415 | 4 | 0 |
| 204320_at | NM_001854 | COL11A1 | 3,4717 | 0,0195 | 4 | 0 |
| 201344_at | BF196642 | UBE2D2 | 3,4904 | 0,0413 | 4 | 0 |
| 211776_s_at | BC006141 | EPB41L3 | 3,5026 | 0,0371 | 4 | 0 |
| 221729_at | AL575735 | COL5A2 | 3,5107 | 0,0412 | 4 | 1 |
| 219099_at | NM_020375 | C12orf5 | 3,5339 | 0,048 | 4 | 0 |
| 201695_s_at | NM_000270 | NP | 3,5608 | 0,0306 | 4 | 0 |
| 218585_s_at | NM_016448 | DTL | 3,5863 | 0,0193 | 4 | 0 |
| 201291_s_at | AU159942 | TOP2A | 3,6575 | 0,01 | 4 | 0 |
| 210511_s_at | M13436 | INHBA | 3,7175 | 0,0179 | 4 | 0 |
| 215646_s_at | R94644 | CSPG2 | 3,7216 | 0,0169 | 4 | 1 |
| 229068_at | BF197357 | CCT5 | 3,7232 | 0,018 | 4 | 1 |
| 228273_at | BG165011 | PRR11 | 3,7356 | 0,0195 | 4 | 0 |
| 235476_at | AW182459 | TRIM59 | 3,7867 | 0,0197 | 5 | 0 |
| 203715_at | NM_003193 | TBCE | 3,8525 | 0,016 | 5 | 0 |
| 211379_x_at | AB050855 | B3GALT3 | 3,8573 | 0,0163 | 4 | 0 |
| 205941_s_at | AI376003 | COL10A1 | 3,8727 | 0,007 | 4 | 0 |
| 216766_at | AK025152 | PRKCE | 3,9817 | 0,0181 | 4 | 0 |
| 209524_at | AK001280 | HDGFRP3 | 4,0233 | 0,018 | 4 | 0 |
| 203102_s_at | NM_002408 | MGAT2 | 4,0953 | 0,0203 | 4 | 0 |
| 211161_s_at | AF130082 | COL3A1 | 4,1092 | 0,0196 | 4 | 0 |
| 235318_at | AW955612 | FBN1 | 4,1413 | 0,0157 | 4 | 0 |
| 211764_s_at | BC005980 | UBE2D1 | 4,1443 | 0,0197 | 4 | 0 |
| 1556499_s_at | BE221212 | COL1A1 | 4,2323 | 0,0163 | 4 | 0 |
| 214590_s_at | AL545760 | UBE2D1 | 4,3105 | 0,0157 | 4 | 0 |
| 218039_at | NM_016359 | NUSAP1 | 4,4104 | 0,0072 | 5 | 0 |
| 214943_s_at | D38491 | RBM34 | 4,4138 | 0,0149 | 4 | 0 |
| 235113_at | AA742244 | PPIL5 | 4,4141 | 0,0139 | 4 | 0 |
| 212353_at | AI479175 | SULF1 | 4,5601 | 0,002 | 4 | 0 |
| 219918_s_at | NM_018123 | ASPM | 4,5827 | 0,0071 | 4 | 0 |
| 218986_s_at | NM_017631 | FLJ20035 | 4,744 | 0,0103 | 4 | 0 |
| 207165_at | NM_012485 | HMMR | 4,8035 | 0,0055 | 4 | 0 |
| 212930_at | AW576457 | ATP2B1 | 4,9577 | 0,0072 | 4 | 0 |
| 217735_s_at | AW007368 | EIF2AK1 | 5,0124 | 0,0053 | 4 | 0 |
| 212354_at | BE500977 | SULF1 | 5,0187 | 0,0063 | 5 | 0 |
| 202310_s_at | K01228 | COL1A1 | 5,0947 | 0,0036 | 4 | 0 |
| 226682_at | AW006185 | LOC283666 | 5,1265 | 0,0069 | 4 | 0 |
| 225681_at | AA584310 | CTHRC1 | 5,1547 | 0,0033 | 4 | 0 |
| 212464_s_at | X02761 | FN1 | 5,2104 | 0,006 | 4 | 0 |
| 224496_s_at | BC006292 | MGC10744 | 5,2348 | 0,005 | 4 | 0 |
| 225706_at | AI761989 | GLCCI1 | 5,3801 | 0,0031 | 5 | 0 |
| 238681_at | R46180 | GDPD1 | 5,4661 | 0,0018 | 4 | 0 |
| 1563321_s_at | AF272384 | MLLT10 | 5,5266 | 0,0025 | 4 | 0 |
| 202311_s_at | AI743621 | COL1A1 | 5,6126 | 0,0017 | 5 | 0 |
| 1567458_s_at | AJ012502 | RAC1 | 5,6203 | 0,002 | 4 | 0 |
| 213909_at | AU147799 | LRRC15 | 6,1642 | 0 | 4 | 0 |
| 37892_at | J04177 | COL11A1 | 6,6985 | 0 | 5 | 0 |
| 202404_s_at | NM_000089 | COL1A2 | 6,763 | 0 | 4 | 0 |
| 205509_at | NM_001871 | CPB1 | 12,5197 | 0 | 4 | 0 |

*FC, fold change for the specified comparison by rank products analysis; pfp, percentage of false positivities in rank products analysis; Increase – Decrease, count of changes more than 4 (out of 5) was used for parwise comparisons between tumour and normal cells.*

Supplementary Table 4. Differentially expressed probe sets between ductal (Tduc) and lobular carcinoma (Tlob) cells found by both rank products and pairwise analysis.

| Probe set | Accession | Gene Symbol | FC:(Tduc/Tlob) | pfp | Increase | Decrease |
| --- | --- | --- | --- | --- | --- | --- |
| 219768_at | NM_024626 | VTCN1 | -7,8064 | 0 | 2 | 21 |
| 220770_s_at | NM_022090 | LOC63920 | -5,3135 | 0,021 | 0 | 24 |
| 214657_s_at | AU134977 | --- | -4,51264 | 0,071 | 0 | 21 |
| 203637_s_at | NM_000381 | MID1 | -3,98089 | 0,09 | 0 | 20 |
| 219087_at | NM_017680 | ASPN | -3,85208 | 0,0878 | 0 | 23 |
| 204041_at | NM_000898 | MAOB | -3,54233 | 0,0824 | 4 | 20 |
| 212902_at | BE645231 | SEC24A | -3,48068 | 0,0952 | 2 | 19 |
| 208576_s_at | NM_003537 | HIST1H3B | 3,3482 | 0,0909 | 19 | 1 |
| 225177_at | AA143793 | RAB11FIP1 | 3,483 | 0,0915 | 21 | 2 |
| 229366_at | BG149765 | CRBN | 3,5567 | 0,0775 | 20 | 0 |
| 230534_at | AW025362 | MGC15634 | 3,5981 | 0,0783 | 20 | 2 |
| 228069_at | AL138828 | FAM54A | 3,7975 | 0,0746 | 20 | 0 |
| 226671_at | AI150000 | --- | 3,8245 | 0,0988 | 20 | 2 |
| 226115_at | AI138934 | AHCTF1 | 3,9484 | 0,0923 | 22 | 0 |
| 219073_s_at | NM_017784 | OSBPL10 | 3,9724 | 0,0795 | 21 | 1 |
| 211379_x_at | AB050855 | B3GALT3 | 4,0264 | 0,0648 | 20 | 0 |
| 1553979_at | BC020854 | --- | 4,1034 | 0,0651 | 19 | 0 |
| 225491_at | AL157452 | SLC1A2 | 4,1113 | 0,0615 | 20 | 0 |
| 241863_x_at | AA703326 | TTC14 | 4,3824 | 0,0597 | 20 | 1 |
| 223746_at | BC005231 | STK4 | 4,4682 | 0,0597 | 20 | 2 |
| 225706_at | AI761989 | GLCCI1 | 4,4735 | 0,0659 | 25 | 0 |
| 1569472_s_at | BC026260 | TTC3 | 4,4945 | 0,0626 | 23 | 0 |
| 218247_s_at | NM_016626 | RKHD2 | 4,595 | 0,0727 | 19 | 0 |
| 218585_s_at | NM_016448 | DTL | 4,6388 | 0,0362 | 19 | 1 |
| 204170_s_at | NM_001827 | CKS2 | 4,8428 | 0,0425 | 20 | 2 |
| 226067_at | AL355392 | C20orf114 | 4,914 | 0,0336 | 19 | 0 |
| 1568838_at | AI015847 | --- | 4,9579 | 0,0314 | 20 | 1 |
| 215392_at | AU148154 | MINPP1 | 4,9988 | 0,0376 | 21 | 1 |
| 211776_s_at | BC006141 | EPB41L3 | 5,2622 | 0,0361 | 21 | 0 |
| 204351_at | NM_005980 | S100P | 5,2937 | 0,0275 | 19 | 0 |
| 201131_s_at | NM_004360 | CDH1 | 5,9657 | 0,005 | 19 | 3 |
| 205509_at | NM_001871 | CPB1 | 11,0715 | 0 | 19 | 1 |

*FC, fold change for the specified comparison by rank products analysis; pfp, percentage of false positivities in rank products analysis; Increase – Decrease, count of changes more than 19 (out of 25 inter-patient comparisons - every ductal carcinoma against every lobular carcinoma) was used for comparisons of tumour cells in between.*

Supplementary Table 5. Differentially expressed probe sets between lobular carcinoma (Tlob) and normal ductal (D) cells found by both rank products and pairwise analysis.

| Probe Set | Accession | Gene Symbol | FC:(Tlob/ normal ductal) | pfp | Increase | Decrease |
| --- | --- | --- | --- | --- | --- | --- |
| 209351_at | BC002690 | KRT14 | -23,9808 | 0 | 0 | 4 |
| 204455_at | NM_001723 | DST | -18,2815 | 0 | 0 | 5 |
| 229947_at | AI088609 | PI15 | -15,0376 | 0 | 0 | 5 |
| 205157_s_at | NM_000422 | KRT17 | -12,6263 | 0 | 0 | 4 |
| 214087_s_at | BF593509 | MYBPC1 | -12,21 | 0 | 1 | 4 |
| 201820_at | NM_000424 | KRT5 | -12,1065 | 0 | 0 | 5 |
| 206325_at | NM_001756 | SERPINA6 | -11,3379 | 0 | 0 | 4 |
| 212236_x_at | Z19574 | KRT17 | -8,76424 | 0,0006 | 0 | 4 |
| 209301_at | M36532 | CA2 | -8,68056 | 0,0008 | 0 | 4 |
| 205044_at | NM_014211 | GABRP | -8,59107 | 0,0008 | 0 | 4 |
| 220625_s_at | AF115403 | ELF5 | -8,41751 | 0,0006 | 0 | 5 |
| 227742_at | AI638295 | CLIC6 | -8,23723 | 0,0009 | 0 | 4 |
| 225817_at | AB051536 | CGNL1 | -7,83085 | 0,0028 | 0 | 5 |
| 204734_at | NM_002275 | KRT15 | -7,82473 | 0,002 | 0 | 4 |
| 212730_at | AK026420 | DMN | -7,49625 | 0,0021 | 1 | 4 |
| 205259_at | NM_000901 | NR3C2 | -7,37463 | 0,0022 | 0 | 4 |
| 218824_at | NM_018215 | FLJ10781 | -7,26744 | 0,0056 | 0 | 5 |
| 203951_at | NM_001299 | CNN1 | -7,26216 | 0,0021 | 0 | 4 |
| 202037_s_at | NM_003012 | SFRP1 | -7,25689 | 0,0043 | 1 | 4 |
| 201131_s_at | NM_004360 | CDH1 | -7,21501 | 0,0017 | 0 | 5 |
| 222862_s_at | BG169832 | AK5 | -6,78887 | 0,0021 | 0 | 5 |
| 236835_at | AI654093 | FUT8 | -6,65336 | 0,01 | 0 | 4 |
| 205380_at | NM_002614 | PDZK1 | -6,37755 | 0,0055 | 0 | 5 |
| 206509_at | NM_002652 | PIP | -6,32911 | 0,002 | 1 | 4 |
| 219850_s_at | NM_012153 | EHF | -6,31313 | 0,0094 | 0 | 5 |
| 222196_at | AK000470 | LOC286434 | -6,3012 | 0,01 | 0 | 4 |
| 227314_at | N95414 | ITGA2 | -6,19195 | 0,0123 | 0 | 4 |
| 203397_s_at | BF063271 | GALNT3 | -6,03865 | 0,0155 | 0 | 5 |
| 209291_at | AW157094 | ID4 | -6,03865 | 0,0102 | 0 | 4 |
| 234982_at | BF577193 | ZNF650 | -5,84112 | 0,0139 | 0 | 5 |
| 205073_at | NM_000775 | CYP2J2 | -5,8072 | 0,0158 | 0 | 5 |
| 230061_at | AW338625 | TM4SF18 | -5,64334 | 0,0172 | 1 | 4 |
| 226594_at | AA528157 | ENTPD5 | -5,61798 | 0,0259 | 0 | 4 |
| 204400_at | NM_005864 | EFS | -5,5991 | 0,028 | 0 | 4 |
| 202274_at | NM_001615 | ACTG2 | -5,57414 | 0,0151 | 0 | 4 |
| 217744_s_at | NM_022121 | PERP | -5,51268 | 0,0126 | 0 | 5 |
| 227239_at | AV734839 | DRCTNNB1A | -5,50358 | 0,0253 | 1 | 4 |
| 224823_at | AA526844 | MYLK | -5,42594 | 0,0188 | 0 | 4 |
| 225368_at | BF218115 | HIPK2 | -5,33333 | 0,0325 | 1 | 4 |
| 202131_s_at | NM_003831 | RIOK3 | -5,33049 | 0,0418 | 0 | 4 |
| 212536_at | AB023173 | ATP11B | -5,2687 | 0,0368 | 0 | 4 |
| 230472_at | AI870306 | IRX1 | -5,14668 | 0,0181 | 0 | 4 |
| 229699_at | AW237752 | --- | -5,10465 | 0,0257 | 0 | 5 |
| 238750_at | AW083576 | CCL28 | -5,06842 | 0,026 | 0 | 4 |
| 215300_s_at | AK022172 | FMO5 | -5,04541 | 0,0337 | 0 | 4 |
| 219343_at | NM_017913 | CDC37L1 | -5 | 0,0318 | 0 | 5 |
| 213680_at | AI831452 | KRT6B | -4,99251 | 0,0183 | 0 | 4 |
| 203881_s_at | NM_004010 | DMD | -4,93583 | 0,032 | 0 | 4 |
| 1559739_at | AK025141 | CHPT1 | -4,92126 | 0,042 | 0 | 4 |
| 227764_at | AA227842 | MGC52057 | -4,87567 | 0,0254 | 0 | 5 |
| 222035_s_at | AI984479 | PAPOLA | -4,84262 | 0,0417 | 1 | 4 |
| 227702_at | AA557324 | CYP4X1 | -4,81928 | 0,018 | 1 | 4 |
| 823_at | U84487 | CX3CL1 | -4,80769 | 0,0422 | 0 | 4 |
| 203373_at | NM_003877 | SOCS2 | -4,76872 | 0,0513 | 1 | 4 |
| 1557543_at | AL832672 | NOTCH2 | -4,58295 | 0,0481 | 0 | 4 |
| 239250_at | BE966038 | ZNF542 | -4,55581 | 0,0455 | 0 | 4 |
| 207938_at | NM_015886 | PI15 | -4,51264 | 0,0323 | 0 | 5 |
| 224189_x_at | AF124438 | EHF | -4,46229 | 0,0422 | 0 | 5 |
| 203439_s_at | BC000658 | STC2 | -4,45236 | 0,0253 | 1 | 4 |
| 209292_at | AL022726 | ID4 | -4,45038 | 0,0185 | 1 | 4 |
| 221958_s_at | AA775681 | C1orf139 | -4,44444 | 0,0588 | 0 | 4 |
| 225207_at | AV707102 | PDK4 | -4,44247 | 0,0464 | 1 | 4 |
| 221872_at | AI669229 | RARRES1 | -4,4287 | 0,0171 | 1 | 4 |
| 203438_at | AI435828 | STC2 | -4,42674 | 0,0276 | 0 | 4 |
| 219115_s_at | NM_014432 | IL20RA | -4,26985 | 0,0455 | 0 | 5 |
| 202746_at | AL021786 | ITM2A | -4,26076 | 0,0536 | 0 | 4 |
| 217014_s_at | AC004522 | AZGP1 | -4,24628 | 0,0451 | 0 | 5 |
| 221796_at | AA707199 | NTRK2 | -4,24628 | 0,0161 | 1 | 4 |
| 205898_at | U20350 | CX3CR1 | -4,24268 | 0,0435 | 0 | 4 |
| 220230_s_at | NM_016229 | CYB5R2 | -4,23012 | 0,051 | 0 | 4 |
| 223099_s_at | BC004234 | LONPL | -4,2123 | 0,0723 | 0 | 4 |
| 224361_s_at | AF250309 | IL17RB | -4,19815 | 0,0459 | 1 | 4 |
| 225645_at | AI763378 | EHF | -4,18585 | 0,0423 | 0 | 5 |
| 1557240_a_at | BU689085 | BBX | -4,1684 | 0,048 | 1 | 4 |
| 225297_at | AV715391 | CCDC5 | -4,1632 | 0,0539 | 0 | 5 |
| 209270_at | L25541 | LAMB3 | -4,14766 | 0,0467 | 1 | 4 |
| 227253_at | AI922198 | CP | -4,14079 | 0,0309 | 1 | 4 |
| 1557239_at | BU689085 | BBX | -4,12201 | 0,0433 | 1 | 4 |
| 235182_at | AI816793 | C20orf82 | -4,12031 | 0,0551 | 0 | 4 |
| 227017_at | BE644894 | ERICH1 | -4,10341 | 0,0745 | 0 | 4 |
| 227312_at | AI694536 | --- | -4,05351 | 0,0455 | 0 | 4 |
| 243140_at | AI917901 | ACTA2 | -4,03063 | 0,018 | 1 | 4 |
| 222829_s_at | BE219979 | IL20RA | -3,98406 | 0,0669 | 0 | 4 |
| 212560_at | AV728268 | C11orf32 | -3,97931 | 0,0666 | 0 | 5 |
| 235513_at | AW131450 | --- | -3,96511 | 0,0673 | 0 | 4 |
| 208703_s_at | BG427393 | APLP2 | -3,92311 | 0,0962 | 0 | 4 |
| 224873_s_at | AK024433 | MRPS25 | -3,91389 | 0,0784 | 0 | 4 |
| 230763_at | AA905508 | LOC128153 | -3,85208 | 0,0824 | 0 | 4 |
| 58916_at | AI672101 | KCTD14 | -3,81388 | 0,0823 | 0 | 4 |
| 209309_at | D90427 | AZGP1 | -3,80228 | 0,0734 | 0 | 5 |
| 219255_x_at | NM_018725 | IL17RB | -3,75094 | 0,0677 | 1 | 4 |
| 224156_x_at | AF208111 | IL17RB | -3,74532 | 0,0705 | 1 | 4 |
| 238481_at | AW512787 | MGP | -3,72717 | 0,059 | 1 | 4 |
| 206392_s_at | NM_002888 | RARRES1 | -3,67918 | 0,0631 | 0 | 4 |
| 232361_s_at | AA565141 | EHF | -3,66435 | 0,083 | 0 | 5 |
| 230986_at | AI821447 | KLF8 | -3,6483 | 0,0789 | 0 | 4 |
| 1560850_at | BC016831 | --- | -3,61925 | 0,0798 | 0 | 4 |
| 204259_at | NM_002423 | MMP7 | -3,60881 | 0,0701 | 1 | 4 |
| 202719_s_at | BC001451 | TES | -3,58295 | 0,0904 | 0 | 5 |
| 225386_s_at | AI559701 | HNRPLL | -3,57526 | 0,0417 | 0 | 4 |
| 205509_at | NM_001871 | CPB1 | -3,57398 | 0,048 | 1 | 4 |
| 1556666_a_at | BU680030 | TTC6 | -3,56761 | 0,0215 | 0 | 4 |
| 218963_s_at | NM_015515 | KRT23 | -3,49284 | 0,0792 | 0 | 5 |
| 216918_s_at | AL096710 | DST | -3,49284 | 0,0981 | 0 | 5 |
| 204897_at | AA897516 | PTGER4 | -3,49162 | 0,0944 | 1 | 4 |
| 237839_at | BF433975 | ANK3 | -3,46981 | 0,0979 | 0 | 4 |
| 202504_at | NM_012101 | TRIM29 | -3,45304 | 0,0789 | 0 | 4 |
| 207144_s_at | NM_004143 | CITED1 | -3,44828 | 0,0901 | 1 | 4 |
| 206825_at | NM_000916 | OXTR | -3,36927 | 0,067 | 1 | 4 |
| 224240_s_at | AF266504 | CCL28 | -3,34784 | 0,0725 | 0 | 5 |
| 225792_at | AA618420 | HOOK1 | -3,26584 | 0,0744 | 0 | 4 |
| 212741_at | AA923354 | MAOA | -3,01296 | 0,0902 | 0 | 4 |
| 214456_x_at | M23699 | SAA1 | -2,93686 | 0,0993 | 0 | 4 |
| 215446_s_at | L16895 | LOX | 3,2337 | 0,0997 | 4 | 1 |
| 202988_s_at | NM_002922 | RGS1 | 3,3251 | 0,089 | 4 | 1 |
| 212353_at | AI479175 | SULF1 | 3,3382 | 0,0896 | 4 | 0 |
| 209795_at | L07555 | CD69 | 3,3992 | 0,0989 | 4 | 0 |
| 213869_x_at | AA218868 | THY1 | 3,5095 | 0,0938 | 5 | 0 |
| 213790_at | W46291 | ADAM12 | 3,5356 | 0,0802 | 4 | 1 |
| 232458_at | AU146808 | --- | 3,541 | 0,0867 | 4 | 1 |
| 228141_at | AA173223 | NUDT4 | 3,5543 | 0,0756 | 4 | 0 |
| 1558747_at | AA336502 | SMCHD1 | 3,5712 | 0,0723 | 4 | 0 |
| 210511_s_at | M13436 | INHBA | 3,5953 | 0,0962 | 4 | 1 |
| 212488_at | N30339 | COL5A1 | 3,6056 | 0,0703 | 4 | 0 |
| 204844_at | L12468 | ENPEP | 3,6736 | 0,0441 | 4 | 0 |
| 204468_s_at | NM_005424 | TIE1 | 3,7066 | 0,0413 | 4 | 0 |
| 212764_at | AI806174 | --- | 3,7517 | 0,0502 | 4 | 0 |
| 225664_at | AA788946 | COL12A1 | 3,7751 | 0,0901 | 5 | 0 |
| 217028_at | AJ224869 | CXCR4 | 3,7885 | 0,0961 | 4 | 0 |
| 219158_s_at | NM_025085 | NARG1 | 3,8403 | 0,0443 | 4 | 1 |
| 206227_at | NM_003613 | CILP | 3,8874 | 0,0774 | 4 | 0 |
| 1555971_s_at | AU154086 | FBXO28 | 3,9212 | 0,0904 | 5 | 0 |
| 200890_s_at | AW006345 | SSR1 | 3,9423 | 0,0952 | 4 | 0 |
| 218009_s_at | NM_003981 | PRC1 | 3,9455 | 0,0855 | 4 | 0 |
| 216050_at | AK024584 | --- | 3,9605 | 0,0676 | 4 | 0 |
| 213418_at | NM_002155 | HSPA6 | 4,058 | 0,0778 | 5 | 0 |
| 202664_at | AW058622 | WASPIP | 4,0941 | 0,0764 | 5 | 0 |
| 243496_at | AW367507 | --- | 4,0999 | 0,062 | 5 | 0 |
| 212468_at | AK023512 | SPAG9 | 4,1003 | 0,0861 | 4 | 1 |
| 226695_at | AA775472 | PRRX1 | 4,2042 | 0,0508 | 5 | 0 |
| 214453_s_at | NM_006417 | IFI44 | 4,2169 | 0,078 | 5 | 0 |
| 225583_at | AL573637 | UXS1 | 4,2905 | 0,0481 | 5 | 0 |
| 224827_at | AK022894 | DC-UbP | 4,2987 | 0,0708 | 4 | 0 |
| 211651_s_at | M20206 | LAMB1 | 4,3766 | 0,0257 | 4 | 0 |
| 229723_at | BF591040 | TAGAP | 4,3856 | 0,0505 | 5 | 0 |
| 201792_at | NM_001129 | AEBP1 | 4,4789 | 0,0319 | 4 | 0 |
| 224724_at | AL133001 | SULF2 | 4,4928 | 0,0423 | 5 | 0 |
| 204500_s_at | NM_015239 | AGTPBP1 | 4,5273 | 0,0593 | 4 | 0 |
| 224791_at | AW513835 | DDEF1 | 4,5643 | 0,0223 | 4 | 0 |
| 207117_at | NM_015852 | H-plk | 4,6229 | 0,0111 | 4 | 0 |
| 209596_at | AF245505 | MXRA5 | 4,6759 | 0,0397 | 4 | 0 |
| 241709_s_at | AA599017 | DOCK1 | 4,7087 | 0,0406 | 4 | 0 |
| 220088_at | NM_001736 | C5R1 | 4,8611 | 0,0188 | 4 | 0 |
| 200665_s_at | NM_003118 | SPARC | 4,9385 | 0,0331 | 4 | 0 |
| 227539_at | AW298099 | GNA13 | 4,9461 | 0,0292 | 4 | 0 |
| 219675_s_at | NM_025076 | UXS1 | 4,9467 | 0,0289 | 4 | 0 |
| 212473_s_at | BE965029 | MICAL2 | 4,9712 | 0,021 | 5 | 0 |
| 201261_x_at | BC002416 | BGN | 4,9788 | 0,0167 | 4 | 0 |
| 219700_at | NM_020405 | PLXDC1 | 4,9835 | 0,04 | 4 | 1 |
| 1561760_s_at | AF085995 | LOC441038 | 5,1342 | 0,0204 | 4 | 1 |
| 210809_s_at | D13665 | POSTN | 5,1745 | 0,0206 | 5 | 0 |
| 213241_at | AF035307 | PLXNC1 | 5,1967 | 0,0113 | 4 | 1 |
| 1556499_s_at | BE221212 | COL1A1 | 5,2833 | 0,0172 | 4 | 0 |
| 1556474_a_at | AK095698 | FLJ38379 | 5,3124 | 0,0075 | 4 | 0 |
| 226777_at | AA147933 | ADAM12 | 5,3188 | 0,0204 | 5 | 0 |
| 1555778_a_at | AY140646 | POSTN | 5,4705 | 0,0097 | 5 | 0 |
| 218668_s_at | NM_021183 | RAP2C | 5,5643 | 0,0117 | 4 | 0 |
| 204620_s_at | NM_004385 | CSPG2 | 5,8082 | 0,0089 | 5 | 0 |
| 37892_at | J04177 | COL11A1 | 5,8644 | 0,0093 | 4 | 0 |
| 226237_at | AL359062 | COL8A1 | 5,8754 | 0,01 | 4 | 0 |
| 204463_s_at | AU118882 | EDNRA | 5,9081 | 0,0037 | 4 | 1 |
| 235318_at | AW955612 | FBN1 | 5,9684 | 0,0095 | 4 | 1 |
| 221731_x_at | BF218922 | CSPG2 | 6,1199 | 0,0073 | 5 | 0 |
| 203083_at | NM_003247 | THBS2 | 6,4687 | 0,0079 | 4 | 0 |
| 209541_at | AI972496 | IGF1 | 6,5395 | 0,0041 | 4 | 1 |
| 202403_s_at | AA788711 | COL1A2 | 7,0173 | 0,0038 | 4 | 0 |
| 226930_at | AI345957 | FNDC1 | 7,0557 | 0,0037 | 4 | 0 |
| 223122_s_at | AF311912 | SFRP2 | 7,1332 | 0,0036 | 4 | 1 |
| 213905_x_at | AA845258 | BGN /// SDCCAG33 | 7,2009 | 0,0013 | 5 | 0 |
| 213975_s_at | AV711904 | LYZ /// LILRB1 | 7,3459 | 0,0016 | 5 | 0 |
| 223121_s_at | AW003584 | SFRP2 | 7,3872 | 0,0038 | 4 | 0 |
| 201438_at | NM_004369 | COL6A3 | 7,737 | 0,0015 | 5 | 0 |
| 205713_s_at | NM_000095 | COMP | 8,1414 | 0,0014 | 4 | 0 |
| 221730_at | NM_000393 | COL5A2 | 8,3603 | 0,0011 | 5 | 0 |
| 229218_at | AA628535 | COL1A2 | 8,4169 | 0,0013 | 5 | 0 |
| 216442_x_at | AK026737 | FN1 | 9,6642 | 0,0006 | 5 | 0 |
| 210495_x_at | AF130095 | FN1 | 9,895 | 0,0006 | 5 | 0 |
| 212464_s_at | X02761 | FN1 | 10,5108 | 0 | 5 | 0 |
| 202310_s_at | K01228 | COL1A1 | 10,8295 | 0 | 4 | 0 |
| 204619_s_at | BF590263 | CSPG2 | 11,0285 | 0 | 4 | 0 |
| 221729_at | AL575735 | COL5A2 | 11,2716 | 0 | 5 | 0 |
| 213909_at | AU147799 | LRRC15 | 11,7882 | 0 | 4 | 0 |
| 229802_at | AA147884 | WISP1 | 12,3532 | 0 | 4 | 0 |
| 211719_x_at | BC005858 | FN1 | 12,5355 | 0 | 5 | 0 |
| 202311_s_at | AI743621 | COL1A1 | 12,951 | 0 | 5 | 0 |
| 201852_x_at | AI813758 | COL3A1 | 13,7334 | 0 | 4 | 0 |
| 215076_s_at | AU144167 | COL3A1 | 13,8233 | 0 | 5 | 0 |
| 211161_s_at | AF130082 | COL3A1 | 14,933 | 0 | 5 | 0 |
| 202404_s_at | NM_000089 | COL1A2 | 18,3294 | 0 | 5 | 0 |
| 225681_at | AA584310 | CTHRC1 | 18,4965 | 0 | 5 | 0 |
| 219087_at | NM_017680 | ASPN | 23,2815 | 0 | 5 | 0 |

*FC, fold change for the specified comparison by rank products analysis; pfp, percentage of false positivities in rank products analysis; Increase – Decrease, count of changes more than 4 (out of 5) was used for parwise comparisons between tumour and normal cells.*

Supplementary Table 6. Differentially expressed probe sets between lobular carcinoma (Tlob) and normal lobular (L) cells found by both rank products and pairwise analysis.

| Probe Set | Accession | Gene Symbol | FC:(Tlob/ normal lobular) | pfp | Increase | Decrease |
| --- | --- | --- | --- | --- | --- | --- |
| 204455_at | NM_001723 | DST | -22,1729 | 0 | 0 | 5 |
| 209351_at | BC002690 | KRT14 | -21,8818 | 0 | 0 | 5 |
| 229947_at | AI088609 | PI15 | -18,5185 | 0 | 0 | 5 |
| 204712_at | NM_007191 | WIF1 | -17,8571 | 0 | 0 | 4 |
| 201820_at | NM_000424 | KRT5 | -11,2613 | 0 | 0 | 5 |
| 209292_at | AL022726 | ID4 | -10,7991 | 0 | 0 | 5 |
| 202833_s_at | NM_000295 | SERPINA1 | -10,2987 | 0 | 0 | 4 |
| 214087_s_at | BF593509 | MYBPC1 | -10,0705 | 0 | 0 | 4 |
| 205157_s_at | NM_000422 | KRT17 | -9,90099 | 0 | 0 | 5 |
| 227742_at | AI638295 | CLIC6 | -9,34579 | 0 | 0 | 5 |
| 209301_at | M36532 | CA2 | -9,19118 | 0 | 0 | 4 |
| 243140_at | AI917901 | ACTA2 | -8,69565 | 0 | 0 | 5 |
| 211429_s_at | AF119873 | SERPINA1 | -8,63558 | 0 | 0 | 4 |
| 202037_s_at | NM_003012 | SFRP1 | -8,47458 | 0 | 0 | 4 |
| 212730_at | AK026420 | DMN | -7,20981 | 0,0033 | 0 | 5 |
| 202342_s_at | NM_015271 | TRIM2 | -7,11238 | 0,0033 | 0 | 5 |
| 206509_at | NM_002652 | PIP | -7,01262 | 0,0025 | 0 | 4 |
| 212236_x_at | Z19574 | KRT17 | -6,98812 | 0,0035 | 0 | 5 |
| 203951_at | NM_001299 | CNN1 | -6,93481 | 0,0037 | 0 | 5 |
| 229357_at | BF060767 | ADAMTS5 | -6,64011 | 0,0081 | 1 | 4 |
| 204734_at | NM_002275 | KRT15 | -6,36132 | 0,0039 | 0 | 4 |
| 201131_s_at | NM_004360 | CDH1 | -6,2461 | 0,0036 | 0 | 4 |
| 205044_at | NM_014211 | GABRP | -6,16523 | 0,0032 | 1 | 4 |
| 224823_at | AA526844 | MYLK | -5,94884 | 0,0117 | 0 | 4 |
| 242374_at | AA747563 | HIAT1 | -5,90319 | 0,0135 | 0 | 4 |
| 225817_at | AB051536 | CGNL1 | -5,88235 | 0,0139 | 0 | 4 |
| 220625_s_at | AF115403 | ELF5 | -5,87889 | 0,0061 | 0 | 5 |
| 205358_at | NM_000826 | GRIA2 | -5,7241 | 0,0024 | 1 | 4 |
| 205413_at | NM_001584 | MPPED2 | -5,59284 | 0,0116 | 0 | 4 |
| 206825_at | NM_000916 | OXTR | -5,46448 | 0,0142 | 0 | 5 |
| 226147_s_at | AA838075 | PIGR | -5,39665 | 0,0136 | 0 | 4 |
| 203881_s_at | NM_004010 | DMD | -5,35045 | 0,0113 | 0 | 4 |
| 223412_at | AL136782 | KBTBD7 | -5,25486 | 0,0148 | 0 | 4 |
| 221795_at | AI346341 | NTRK2 | -5,10986 | 0,022 | 0 | 5 |
| 203438_at | AI435828 | STC2 | -5,0226 | 0,0135 | 0 | 4 |
| 215300_s_at | AK022172 | FMO5 | -4,93583 | 0,0322 | 0 | 4 |
| 1557286_at | AK001007 | --- | -4,89476 | 0,0187 | 0 | 4 |
| 221530_s_at | BE857425 | BHLHB3 | -4,84262 | 0,0218 | 0 | 5 |
| 227194_at | BF106962 | FAM3B | -4,82393 | 0,0133 | 0 | 4 |
| 201983_s_at | AW157070 | EGFR | -4,7824 | 0,0322 | 0 | 5 |
| 209270_at | L25541 | LAMB3 | -4,76417 | 0,0219 | 0 | 5 |
| 226753_at | AW138704 | FAM76B | -4,71921 | 0,0402 | 0 | 4 |
| 227314_at | N95414 | ITGA2 | -4,69484 | 0,0324 | 0 | 4 |
| 218247_s_at | NM_016626 | RKHD2 | -4,66636 | 0,0365 | 0 | 4 |
| 209170_s_at | AF016004 | GPM6B | -4,65333 | 0,0348 | 0 | 4 |
| 220324_at | NM_024882 | C6orf155 | -4,55373 | 0,0366 | 0 | 4 |
| 1565162_s_at | D16947 | MGST1 | -4,52694 | 0,0169 | 0 | 4 |
| 216918_s_at | AL096710 | DST | -4,49236 | 0,0319 | 0 | 5 |
| 232528_at | AI338705 | UBE2E3 | -4,48632 | 0,0432 | 1 | 4 |
| 228554_at | AL137566 | --- | -4,48229 | 0,0329 | 0 | 5 |
| 227627_at | AV690866 | SGK3 | -4,47628 | 0,0321 | 0 | 4 |
| 229596_at | AW271617 | MGC35366 | -4,46828 | 0,0449 | 1 | 4 |
| 225297_at | AV715391 | CCDC5 | -4,4287 | 0,0329 | 0 | 4 |
| 203075_at | AW151617 | SMAD2 | -4,37828 | 0,0405 | 0 | 4 |
| 242865_at | AI332638 | NPTN | -4,3573 | 0,0493 | 1 | 4 |
| 241060_x_at | H37807 | TSPAN5 | -4,33088 | 0,0325 | 0 | 4 |
| 209555_s_at | M98399 | CD36 | -4,32526 | 0,0132 | 1 | 4 |
| 220230_s_at | NM_016229 | CYB5R2 | -4,32152 | 0,0318 | 0 | 4 |
| 230061_at | AW338625 | TM4SF18 | -4,29738 | 0,0364 | 1 | 4 |
| 204004_at | AI336206 | PAWR | -4,28449 | 0,0291 | 0 | 4 |
| 205380_at | NM_002614 | PDZK1 | -4,22119 | 0,0112 | 0 | 4 |
| 225645_at | AI763378 | EHF | -4,17188 | 0,0364 | 0 | 4 |
| 226671_at | AI150000 | --- | -4,1425 | 0,0669 | 0 | 4 |
| 213375_s_at | N80918 | CG018 | -4,13907 | 0,0708 | 0 | 4 |
| 226493_at | AI627249 | KCTD18 | -4,12882 | 0,057 | 0 | 5 |
| 217744_s_at | NM_022121 | PERP | -4,12371 | 0,0325 | 1 | 4 |
| 221958_s_at | AA775681 | C1orf139 | -4,11692 | 0,0579 | 1 | 4 |
| 219850_s_at | NM_012153 | EHF | -4,095 | 0,0238 | 0 | 4 |
| 220038_at | NM_013257 | SGK3 | -4,08497 | 0,017 | 0 | 4 |
| 207938_at | NM_015886 | PI15 | -4,07498 | 0,0329 | 0 | 4 |
| 222035_s_at | AI984479 | PAPOLA | -4,07 | 0,065 | 1 | 4 |
| 209512_at | BC004331 | HSDL2 | -4,06835 | 0,0657 | 0 | 4 |
| 205073_at | NM_000775 | CYP2J2 | -4,05351 | 0,057 | 0 | 4 |
| 202504_at | NM_012101 | TRIM29 | -4,03063 | 0,0364 | 0 | 5 |
| 200974_at | NM_001613 | ACTA2 | -4,00641 | 0,0567 | 0 | 5 |
| 201496_x_at | S67238 | MYH11 | -4,00641 | 0,0745 | 0 | 4 |
| 227702_at | AA557324 | CYP4X1 | -4 | 0,0327 | 0 | 4 |
| 1565666_s_at | AW864944 | MUC6 | -3,9984 | 0,019 | 0 | 4 |
| 204485_s_at | NM_005486 | TOM1L1 | -3,98089 | 0,0488 | 0 | 4 |
| 241387_at | AW276701 | PTK2 | -3,96197 | 0,0683 | 0 | 4 |
| 217014_s_at | AC004522 | AZGP1 | -3,9604 | 0,0327 | 0 | 5 |
| 218102_at | NM_015954 | DERA | -3,93856 | 0,0693 | 0 | 4 |
| 1557527_at | BU789637 | --- | -3,93391 | 0,0909 | 0 | 4 |
| 202950_at | NM_001889 | CRYZ | -3,93082 | 0,0854 | 0 | 4 |
| 227764_at | AA227842 | MGC52057 | -3,91696 | 0,0354 | 0 | 4 |
| 207791_s_at | NM_004161 | RAB1A | -3,86548 | 0,096 | 0 | 4 |
| 222156_x_at | AK022459 | CCPG1 | -3,86399 | 0,0541 | 0 | 4 |
| 228854_at | AI492388 | --- | -3,861 | 0,0628 | 0 | 4 |
| 214912_at | AK022067 | --- | -3,80518 | 0,0623 | 0 | 4 |
| 233002_at | AB046842 | KIAA1622 | -3,74953 | 0,0498 | 0 | 4 |
| 203439_s_at | BC000658 | STC2 | -3,74251 | 0,033 | 0 | 4 |
| 208703_s_at | BG427393 | APLP2 | -3,73692 | 0,0914 | 0 | 4 |
| 229955_at | AW772096 | FBXO3 | -3,72995 | 0,0723 | 0 | 4 |
| 222829_s_at | BE219979 | IL20RA | -3,72162 | 0,0682 | 0 | 5 |
| 205509_at | NM_001871 | CPB1 | -3,69276 | 0,0449 | 1 | 4 |
| 228716_at | BG494007 | THRB | -3,69004 | 0,0949 | 0 | 4 |
| 225426_at | AW195360 | PPP6C | -3,65097 | 0,0598 | 0 | 5 |
| 218980_at | NM_025135 | FHOD3 | -3,63769 | 0,0967 | 0 | 4 |
| 244267_at | AA237039 | LOC401056 | -3,63108 | 0,0874 | 1 | 4 |
| 209309_at | D90427 | AZGP1 | -3,62582 | 0,058 | 0 | 5 |
| 209369_at | M63310 | ANXA3 | -3,57015 | 0,0922 | 1 | 4 |
| 219255_x_at | NM_018725 | IL17RB | -3,53357 | 0,0821 | 1 | 4 |
| 222387_s_at | BG476669 | VPS35 | -3,52113 | 0,0486 | 1 | 4 |
| 218184_at | NM_020245 | TULP4 | -3,50018 | 0,096 | 0 | 4 |
| 224361_s_at | AF250309 | IL17RB | -3,48068 | 0,0924 | 1 | 4 |
| 230364_at | BF940025 | CHPT1 | -3,45543 | 0,0687 | 0 | 4 |
| 204591_at | NM_006614 | CHL1 | -3,44471 | 0,0893 | 0 | 4 |
| 233019_at | AU145061 | CNOT7 | -3,43053 | 0,0825 | 0 | 5 |
| 236538_at | BE219628 | GRIA2 | -3,42349 | 0,0238 | 1 | 4 |
| 225207_at | AV707102 | PDK4 | -3,4118 | 0,0814 | 1 | 4 |
| 212560_at | AV728268 | C11orf32 | -3,39443 | 0,0973 | 0 | 5 |
| 225334_at | AI147621 | C10orf32 | -3,37952 | 0,0897 | 1 | 4 |
| 215096_s_at | AU145746 | ESD | -3,35683 | 0,0978 | 0 | 5 |
| 206306_at | NM_001036 | RYR3 | -3,30907 | 0,0839 | 0 | 4 |
| 235182_at | AI816793 | C20orf82 | -3,13578 | 0,0939 | 0 | 4 |
| 239348_at | AI285970 | USP31 | -3,04044 | 0,0846 | 0 | 4 |
| 204955_at | NM_006307 | SRPX | 2,2556 | 0,0921 | 4 | 1 |
| 216834_at | S59049 | RGS1 | 2,7752 | 0,0658 | 4 | 1 |
| 224791_at | AW513835 | DDEF1 | 3,0101 | 0,0973 | 4 | 1 |
| 202988_s_at | NM_002922 | RGS1 | 3,1415 | 0,092 | 4 | 1 |
| 204831_at | R59697 | CDK8 | 3,2477 | 0,0805 | 4 | 1 |
| 232458_at | AU146808 | --- | 3,486 | 0,0716 | 4 | 0 |
| 211981_at | NM_001845 | COL4A1 | 3,5047 | 0,0504 | 4 | 0 |
| 211651_s_at | M20206 | LAMB1 | 3,5562 | 0,0672 | 4 | 1 |
| 222018_at | AI992187 | NACA /// NACAP1 | 3,6708 | 0,0709 | 4 | 1 |
| 74694_s_at | AA907940 | RABEP2 | 3,7179 | 0,0705 | 4 | 1 |
| 231838_at | AK026760 | C20orf119 | 3,7427 | 0,0531 | 4 | 0 |
| 212551_at | NM_006366 | CAP2 | 3,7699 | 0,0922 | 4 | 1 |
| 224724_at | AL133001 | SULF2 | 3,844 | 0,0598 | 5 | 0 |
| 218308_at | NM_006342 | TACC3 | 3,8524 | 0,0882 | 4 | 0 |
| 38241_at | U90548 | BTN3A3 | 3,9002 | 0,0484 | 4 | 0 |
| 223122_s_at | AF311912 | SFRP2 | 3,9076 | 0,0617 | 4 | 1 |
| 202786_at | NM_013233 | STK39 | 3,9119 | 0,0919 | 4 | 0 |
| 214059_at | BE049439 | IFI44 | 3,9179 | 0,0719 | 4 | 0 |
| 212522_at | W73272 | PDE8A | 3,9517 | 0,0749 | 4 | 0 |
| 221731_x_at | BF218922 | CSPG2 | 4,0149 | 0,0477 | 4 | 0 |
| 204051_s_at | AW089415 | SFRP4 | 4,0162 | 0,0362 | 4 | 1 |
| 226695_at | AA775472 | PRRX1 | 4,0237 | 0,05 | 5 | 0 |
| 210809_s_at | D13665 | POSTN | 4,0244 | 0,0472 | 5 | 0 |
| 204776_at | NM_003248 | THBS4 | 4,0395 | 0,0604 | 4 | 0 |
| 213790_at | W46291 | ADAM12 | 4,0401 | 0,0387 | 4 | 0 |
| 201069_at | NM_004530 | MMP2 | 4,054 | 0,0665 | 4 | 0 |
| 209596_at | AF245505 | MXRA5 | 4,0885 | 0,0476 | 5 | 0 |
| 219562_at | NM_014353 | RAB26 | 4,1811 | 0,0654 | 4 | 0 |
| 201792_at | NM_001129 | AEBP1 | 4,1989 | 0,0468 | 4 | 0 |
| 202543_s_at | BC005359 | GMFB | 4,2362 | 0,0476 | 4 | 0 |
| 210114_at | AF039217 | INVS | 4,2404 | 0,0609 | 4 | 0 |
| 218254_s_at | NM_016103 | SAR1B | 4,25 | 0,0368 | 5 | 0 |
| 211964_at | X05610 | COL4A2 | 4,277 | 0,0301 | 5 | 0 |
| 202664_at | AW058622 | WASPIP | 4,3563 | 0,0334 | 4 | 0 |
| 229732_at | AI417785 | HSZFP36 | 4,4573 | 0,0315 | 4 | 0 |
| 201438_at | NM_004369 | COL6A3 | 4,4592 | 0,0319 | 5 | 0 |
| 204620_s_at | NM_004385 | CSPG2 | 4,4781 | 0,0258 | 4 | 0 |
| 220088_at | NM_001736 | C5R1 | 4,4831 | 0,0205 | 4 | 0 |
| 214657_s_at | AU134977 | --- | 4,4862 | 0,043 | 5 | 0 |
| 1556821_x_at | H48516 | --- | 4,5092 | 0,0152 | 4 | 0 |
| 229723_at | BF591040 | TAGAP | 4,511 | 0,0347 | 4 | 0 |
| 1555778_a_at | AY140646 | POSTN | 4,5233 | 0,02 | 5 | 0 |
| 203878_s_at | NM_005940 | MMP11 | 4,5908 | 0,0448 | 4 | 0 |
| 224967_at | W72338 | UGCG | 4,6344 | 0,029 | 4 | 0 |
| 238436_s_at | AV726376 | LOC390980 | 4,6757 | 0,0258 | 4 | 1 |
| 201261_x_at | BC002416 | BGN | 4,8333 | 0,0164 | 4 | 0 |
| 225664_at | AA788946 | COL12A1 | 4,8404 | 0,0282 | 4 | 0 |
| 205269_at | AI123251 | LCP2 | 4,8966 | 0,0102 | 4 | 0 |
| 212646_at | D42043 | RAFTLIN | 5,0135 | 0,01 | 4 | 0 |
| 229861_at | N66669 | LOC440426 | 5,0695 | 0,0149 | 5 | 0 |
| 202589_at | NM_001071 | TYMS | 5,1161 | 0,0208 | 4 | 0 |
| 232165_at | AL137725 | EPPK1 | 5,1858 | 0,0202 | 4 | 0 |
| 1556499_s_at | BE221212 | COL1A1 | 5,336 | 0,0117 | 4 | 0 |
| 227300_at | AL521682 | LOC338773 | 5,3881 | 0,0149 | 5 | 0 |
| 229218_at | AA628535 | COL1A2 | 5,499 | 0,0113 | 5 | 0 |
| 226777_at | AA147933 | ADAM12 | 5,7243 | 0,01 | 5 | 0 |
| 215446_s_at | L16895 | LOX | 5,7418 | 0,0102 | 4 | 0 |
| 219787_s_at | NM_018098 | ECT2 | 5,8072 | 0,0076 | 4 | 0 |
| 223121_s_at | AW003584 | SFRP2 | 5,8153 | 0,0087 | 4 | 1 |
| 202403_s_at | AA788711 | COL1A2 | 6,2089 | 0,0069 | 4 | 0 |
| 209875_s_at | M83248 | SPP1 | 6,4648 | 0,0029 | 5 | 0 |
| 203083_at | NM_003247 | THBS2 | 6,5243 | 0,0054 | 4 | 0 |
| 226930_at | AI345957 | FNDC1 | 6,5399 | 0,0044 | 4 | 1 |
| 215076_s_at | AU144167 | COL3A1 | 6,5671 | 0,0048 | 5 | 0 |
| 37892_at | J04177 | COL11A1 | 6,6394 | 0,0047 | 4 | 0 |
| 205713_s_at | NM_000095 | COMP | 6,6758 | 0,003 | 4 | 0 |
| 217028_at | AJ224869 | CXCR4 | 6,7206 | 0,0028 | 5 | 0 |
| 201852_x_at | AI813758 | COL3A1 | 6,9855 | 0,0029 | 5 | 0 |
| 212489_at | AI983428 | COL5A1 | 7,0685 | 0,0031 | 4 | 0 |
| 212488_at | N30339 | COL5A1 | 7,1614 | 0,0023 | 4 | 0 |
| 213909_at | AU147799 | LRRC15 | 7,3073 | 0,0024 | 4 | 1 |
| 210495_x_at | AF130095 | FN1 | 7,4298 | 0,003 | 5 | 0 |
| 227539_at | AW298099 | GNA13 | 7,5071 | 0,0028 | 4 | 0 |
| 235318_at | AW955612 | FBN1 | 7,6767 | 0,0022 | 4 | 1 |
| 212464_s_at | X02761 | FN1 | 8,0165 | 0,0024 | 5 | 0 |
| 216442_x_at | AK026737 | FN1 | 8,0492 | 0,0025 | 5 | 0 |
| 213905_x_at | AA845258 | BGN /// SDCCAG33 | 8,2684 | 0,002 | 5 | 0 |
| 226237_at | AL359062 | COL8A1 | 8,4093 | 0,0026 | 4 | 1 |
| 204619_s_at | BF590263 | CSPG2 | 8,7529 | 0,0021 | 4 | 0 |
| 217428_s_at | X98568 | COL10A1 | 8,8735 | 0,0028 | 4 | 0 |
| 241342_at | BG288115 | TMEM65 | 9,0614 | 0,0023 | 5 | 0 |
| 229802_at | AA147884 | WISP1 | 9,1643 | 0,0027 | 4 | 0 |
| 221729_at | AL575735 | COL5A2 | 9,3198 | 0,0033 | 5 | 0 |
| 1557411_s_at | AK094254 | LOC203427 | 9,364 | 0,0029 | 5 | 0 |
| 211719_x_at | BC005858 | FN1 | 9,5835 | 0,0025 | 5 | 0 |
| 211161_s_at | AF130082 | COL3A1 | 9,7744 | 0,0025 | 5 | 0 |
| 202310_s_at | K01228 | COL1A1 | 10,3836 | 0,0017 | 5 | 0 |
| 213975_s_at | AV711904 | LYZ /// LILRB1 | 10,7604 | 0 | 5 | 0 |
| 202311_s_at | AI743621 | COL1A1 | 12,6839 | 0 | 5 | 0 |
| 219087_at | NM_017680 | ASPN | 22,1022 | 0 | 5 | 0 |
| 202404_s_at | NM_000089 | COL1A2 | 30,4894 | 0 | 5 | 0 |
| 225681_at | AA584310 | CTHRC1 | 48,3776 | 0 | 5 | 0 |

*FC, fold change for the specified comparison by rank products analysis; pfp, percentage of false positivities in rank products analysis; Increase – Decrease, count of changes more than 4 (out of 5) was used for parwise comparisons between tumour and normal cells.*
